# Single-cell analysis of immune and stroma cell remodeling in clear cell renal cell carcinoma primary tumors and bone metastatic lesions

**DOI:** 10.1186/s13073-023-01272-6

**Published:** 2024-01-29

**Authors:** Shenglin Mei, Adele M. Alchahin, Ioanna Tsea, Youmna Kfoury, Taghreed Hirz, Nathan Elias Jeffries, Ting Zhao, Yanxin Xu, Hanyu Zhang, Hirak Sarkar, Shulin Wu, Alexander O. Subtelny, John Inge Johnsen, Yida Zhang, Keyan Salari, Chin-Lee Wu, Mark A. Randolph, David T. Scadden, Douglas M. Dahl, John Shin, Peter V. Kharchenko, Philip J. Saylor, David B. Sykes, Ninib Baryawno

**Affiliations:** 1 Center for Regenerative Medicine, Massachusetts General Hospital, Boston, MA 02114 USA; 2 Department of Biomedical Informatics, Harvard Medical School, Boston, MA 02115 USA; 3 Childhood Cancer Research Unit, Department of Women’s and Children’s Health, Karolinska Institutet, 17176 Stockholm, Sweden; 4 Harvard Stem Cell Institute, Cambridge, MA 02138 USA; 5 Department of Stem Cell and Regenerative Biology, Harvard University, Cambridge, MA 02138 USA; 6 Department of Pathology, Massachusetts General Hospital, Harvard Medical School, Boston, MA 02114 USA; 7 Department of Urology, Massachusetts General Hospital, Harvard Medical School, Boston, MA 02114 USA; 8 Division of Plastic and Reconstructive Surgery, Massachusetts General Hospital, Boston, MA 02114 USA; 9 Department of Neurosurgery, Harvard Medical School, Boston, MA 02115 USA; 10 Massachusetts General Hospital Cancer Center, Harvard Medical School, Boston, MA 02114 USA; 11 Present: Altos Labs, San Diego, CA 92121 USA

## Abstract

**Background:**

Despite therapeutic advances, once a cancer has metastasized to the bone, it represents a highly morbid and lethal disease. One third of patients with advanced clear cell renal cell carcinoma (ccRCC) present with bone metastasis at the time of diagnosis. However, the bone metastatic niche in humans, including the immune and stromal microenvironments, has not been well-defined, hindering progress towards identification of therapeutic targets.

**Methods:**

We collected fresh patient samples and performed single-cell transcriptomic profiling of solid metastatic tissue (*Bone Met*), liquid bone marrow at the vertebral level of spinal cord compression (*Involved*), and liquid bone marrow from a different vertebral body distant from the tumor site but within the surgical field (*Distal*), as well as bone marrow from patients undergoing hip replacement surgery (*Benign*). In addition, we incorporated single-cell data from primary ccRCC tumors (*ccRCC Primary*) for comparative analysis.

**Results:**

The bone marrow of metastatic patients is immune-suppressive, featuring increased, exhausted CD8 + cytotoxic T cells, T regulatory cells, and tumor-associated macrophages (TAM) with distinct transcriptional states in metastatic lesions. Bone marrow stroma from tumor samples demonstrated a tumor-associated mesenchymal stromal cell population (TA-MSC) that appears to be supportive of epithelial-to mesenchymal transition (EMT), bone remodeling, and a cancer-associated fibroblast (CAFs) phenotype. This stromal subset is associated with poor progression-free and overall survival and also markedly upregulates bone remodeling through the dysregulation of RANK/RANKL/OPG signaling activity in bone cells, ultimately leading to bone resorption.

**Conclusions:**

These results provide a comprehensive analysis of the bone marrow niche in the setting of human metastatic cancer and highlight potential therapeutic targets for both cell populations and communication channels.

**Supplementary Information:**

The online version contains supplementary material available at 10.1186/s13073-023-01272-6.

## Background

Bone metastasis occurs when cancer spreads from the primary tumor site to the bone and bone marrow (BM). Bone metastases represent a common complication of many advanced cancer types and generally portends an incurable disease [1]. While many tumor types can metastasize to the bone, certain cancers have a particular predilection to spread to the bone, including kidney cancer [2]. Clear cell renal cell carcinoma (ccRCC) is a primary kidney cancer that arises from the epithelium of the renal tubule [3]. One third of patients with advanced ccRCC have osteolytic bone metastasis at the time of presentation and a very poor prognosis, with a 5-year survival rate of only 12% [4, 5].

In metastatic cancer, the bone marrow can provide a supportive niche that ultimately allows for colonization of disseminated tumor cells into the microenvironment. Consistent with the “seed and soil” hypothesis, the establishment of bone metastasis likely depends on both cancer cells and the tumor microenvironment (TME). To form metastases, cancer cells overcome the barriers to metastatic spread, giving them a propensity to establish themselves outside their primary tissue [6]. Importantly, when cancer cells arrive and expand in the BM, they can remodel the bone and the BM into a permissive environment favoring tumor cell expansion. Within the normal BM, the growth of disseminated cancer cells should be initially suppressed by macrophages and cytotoxic T cells (CTL) that form an important line of defense against disseminating cancer cells [6]. Unfortunately, changes in the bone metastatic niche can lead to an immunosuppressive TME that disrupts T cell-mediated cell killing and results in an ineffective immune response against the tumor [7].

Most previous work on ccRCC bone metastases has relied on bulk characterization (RNA-sequencing and whole exome/genome sequencing) [8, 9] and has therefore not permitted an in-depth analysis of critical cell type-specific changes of the bone metastatic TME. Many of the cells, molecules, and cell states that make the ccRCC microenvironment, which might be important for tumor growth, remain unknown. Here, we collected fresh human patient from ccRCC primary tumors and bone metastasis lesions for single-cell RNA-seq (scRNA-seq) and provide a comparative single-cell transcriptomic analysis between ccRCC primary tumors and bone metastatic lesions. We defined a distinct tumor-associated macrophage (TAM) population. We demonstrated metastatic specific mesenchymal stromal cells (MSCs) that appear to have the capacity to promote epithelial-to mesenchymal transition (EMT) in tumor cells and that are accompanied by a phenotype of bone remodeling driven by dysregulation of RANK (TNFRSF11A)-RANKL (TNFSF11) and Osteoprotegerin (OPG, TNFRSF11B) signaling.

## Methods

### Patient cohorts and sample collection

ccRCC patients with bone metastatic (*n* = 9) were enrolled in this study. Tumor specimens were submitted to pathology as standard confirmation of diagnosis of ccRCC and bone metastatic disease. The patient was clinically indicated decompression and stabilization in the setting of spinal cord compression related to metastatic ccRCC. The patient was positioned prone under general anesthesia to facilitate posterior spinal access. The insertion of a Jamshidi needle into the osseous structure allowed for the extraction of bone marrow and tumor samples, minimizing the dilution of the specimen with extraneous blood or irrigation fluids present in the surgical field. The aspirate from the vertebral body was then directly collected into sterile tubes, which were promptly transported to the laboratory to undergo further preparative procedures. Similar technique was utilized for the distant vertebral body level samples (e.g., *Distal*). We collect bone metastatic tissue (*Bone Met n* = *9*), liquid bone marrow at the vertebral level of spinal cord compression (*Involved n* = *4*), and liquid bone marrow from a different vertebral body distant from the tumor site but within the surgical field (*Distal n* = *4*) for single-cell transcriptomic profiling. For benign samples, we collect BM samples from patients undergoing hip replacement surgery served as a non-malignant control group (*n* = 9). In total, we generated high-resolution single-cell RNA-Seq profiles from 9 Bone Met samples, 4 Involved BM, 4 Distal BM, 9 Benign BM. The clinical information of all patients was shown in Additional file 1: Table S1.

### Tissue dissociation and cell purification

To dissociate bone metastatic tissues into single cell, all samples were collected in Media 199 supplemented with 2% (v/v) FBS. Single-cell suspensions of the tumors were obtained by cutting the tumor in to small pieces (1 mm^3^) in a 70-mm filter cap, followed by enzymatic dissociation for 45 min at 37 °C with shaking at 120 rpm using Collagenase I, Collagenase II, Collagenase III, Collagenase IV (all at a concentration of 1 mg/ml), and Dispase (2 mg/ml) in the presence of RNase inhibitors (RNasin (Promega) and RNase OUT (Invitrogen). Erythrocytes were subsequently removed by ACK Lysing buffer (Quality Biological) and cells resuspended in Media 199 supplemented with 2% (v/v) FBS for further analysis. For bone marrow aspirate preparation, bone marrow samples were filtered using a 70-micron filter then centrifuged at 600 g for 7 min at 4 °C. Plasma was collected followed by erythrocytes removal using ACK Lysing buffer (Quality Biological). Cells were resuspended in Media 199 supplemented with 2% (v/v) FBS for further analysis.

### FACS sorting

Single cells from tumor and bone marrow samples subjected to RBC lysis were surface stained with anti-*CD235*-PE (Biolegend) for 30 min at 4 °C. Cells were washed twice with 2% FBS-PBS (v/v) followed by DAPI staining (1 µg/ml). For human benign bone marrow stroma samples, bone marrow from hip replacement surgeries was collected in Media199 containing 2%FBS and 12.5 mM EDTA (1:1) volume and strained using 100 µm strainer. The strained BM was enriched using the RosetteSep Human Mesenchymal Stem Cell Enrichment cocktail (Stem cell technologies 15,128) according to manufacturer’s instructions. Bone spicules stuck in the strainer were collected and digested in Media199 containing the following: 2% FBS, RNAse out (Thermo Fisher, 10,777,019), 100 U/ml DNAse (Thermo Fisher 90,083), 2 mg/ml Dispase Gibco, 17,105,041), 1 mg/ml ColI (LS004214), 1 mg/ml ColII (LS004202), 1 mg/ml ColIII (LS004206), 1 mg/ml ColIV (LS004210) all from Worthington Biochemical. Digestion was performed at 37 °C in a shaking water bath at 120 rpm for 45 min. The digestion mix was strained using a 70-µm strainer and rinsed with Media199 + 2%FBS. The cells were counted and enriched for mesenchymal stromal cells using the RosetteSep Human Mesenchymal Stem Cell Enrichment Cocktail (Stem Cell Technologies 15,128) according to manufacturer’s instructions. Cells from both fractions were stained with CD71-PerCpCy5.5, CD235- PerCpCy5.5, CD45-BV711, CD11b-BV711, CD3-BV711, CD19-BV711, CD14-APC, and CD271-PE/Cy7 in Media199 + 2%FBS and RNAse out. Calcein was used for staining the live dead. Flow sorting for live and non-erythroid cells (DAPI-neg/*CD235*-neg) was performed on a BD FACS Aria III equipped with a 100-µm nozzle (BD Biosciences, San Jose, CA) instrument.

### Single-cell RNA-sequencing

All flow cytometry data were analyzed using the FlowJo software (Treestar, San Carlos, CA). Single cells were encapsulated into emulsion droplets using Chromium Controller (10X Genomics). scRNA-seq libraries were constructed using Chromium Single-Cell 3’ v2 Reagent Kit according to the manufacturer’s protocol.

### Summary of scRNA-seq data

To provide an additional comparison, we also analyzed BM single-cell RNA-seq data from healthy individuals published by Oetjen et al. [10]. The data was downloaded from GEO (GSE120221, GSE120446). *ccRCC Primary* tumors and matched *adjacent normal* samples were obtained from our previous study [11]. In total, we have 9 *Bone Met* samples, 4 *Involved* bone marrow, 4 *Distal* bone marrow,12 *Healthy* bone marrow, 9 *Benign* bone marrow, 14 *ccRCC Primary* tumors, and 9 matched *adjacent normal* samples. Detailed sample groups were listed in Additional file 1: Table S1.

### scRNA-Seq data preprocessing and data quality control

Single-cell RNA-seq data were quantified using Cellranger 3.0.2 (10 × Genomics) with reference genome GRCh38. For human benign bone marrow stroma (FASC) samples, we removed non-stroma cells. To remove low-quality and doublets cells, we excluded the following cells: (1) cells with fewer than 700 total UMI, (2) cells with more than 20% mitochondrial transcripts, (3) Scrublet scores above 0.4 using Scrublet (v0.2.3) [12]. Detailed sample and single-cell information was listed in Additional file 1: Table S2 and Table S3.

### scRNA-Seq data processing and batch effect correction

We performed dimensionality reduction and clustering using the Pagoda (v1.0.11) [13] package. Briefly, we first selected top 2000 highly variable genes based on dispersion of variance to mean expression ratios using the pagoda. We then performed principal component analysis (PCA) and reduced the data to the top 30 PCs. The PCA-reduced data were then used to compute a shared nearest neighbor graph, and were further subjected to graph-based clustering with the Louvain Method. To correct batch effects, we used the Conos (v1.5.0) [14] alignment method for data integration. Briefly, these Pagoda2 objects were used to perform alignment with Conos [14], using graph parameters *k* = 20, k.self = 5, space = ‘PCA’, ncomps = 30, n.odgenes = 2000, matching.method = ‘mNN’, and metric = ‘angular’. The graph embedding was estimated using UMAP with default parameters. Leiden clustering was used to determine joint cell clusters across the entire dataset collection. To ensure the robustness of our data integration, we also analyze the data using Seurat (v4.3.0) pipeline [15]. In the Seurat pre-processing pipeline, the NormalizeData and ScaleData functions were applied to obtain comparable expression values, while FindVariableFeatures was employed to identify genes with significant variability across cellular transcriptomic profiles. Additionally, RunPCA, FindNeighbors, FindClusters, and RunUMAP were utilized to calculate reduced-dimension coordinates for visualization and unsupervised clustering. Integration of RCC primary and bone metastasis tumor showed embedding and clustering consistent with Conos results (Additional file 2: Fig. S1C).

### Cell type annotation

**Fig. 1:**
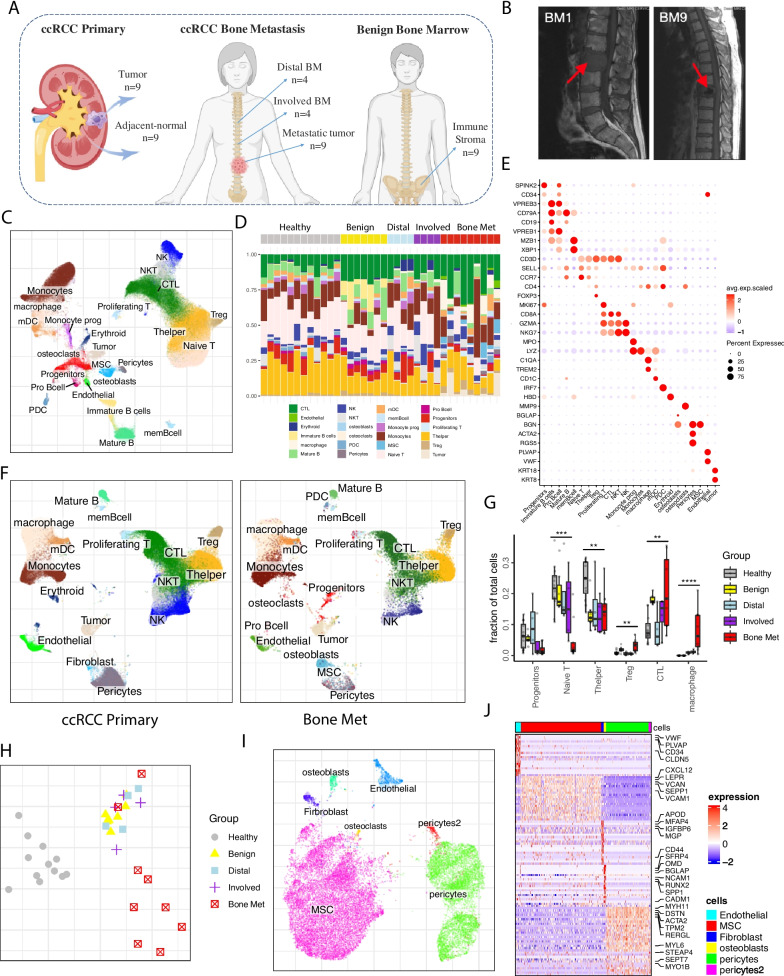
Overview of immune and stromal cell landscape in ccRCC bone metastasis. **A** Schematic illustration of experiment design and patient sample processing. **B** Sagittal T1 MRI imaging of the thoracic spine showing tumor masses with spinal cord compression for BM1 and BM9. **C** Integrative analysis of scRNA-seq samples of all bone marrow samples (*Healthy*, *Benign*, *Involved*, *Distal*, and *Bone Met*), visualized using a common UMAP embedding. **D** Bar plot representing the fraction of major cell types within each sample (column). **E** Dot plot representing key-marker gene expression in major cell types. The color represents scaled average expression of marker genes in each cell type, and the size indicates the proportion of cells expressing marker genes. **F** Integrative analysis of scRNA-seq samples from ccRCC primary and bone metastatic tumors, visualized using a common UMAP embedding for ccRCC primary samples (left), bone metastasis samples (right). **G** Comparison of relative cell abundance of major cell clusters between *Bone Met* (*n* = 9) and different control fractions (*Healthy n* = 12, *Benign n* = 7, *Involved n* = 4, *Distal n* = 4). Statistics are accessed with two-sided Wilcoxon rank sum test and BH multiple testing correction. (*p < 0.05, ***p < 0.001). **H** Pairwise expression distances between samples are shown using MDS embeddings. The similarity measures the magnitude of expression change for each subpopulation, using size-weighted average to combine them into an overall expression distance that controls the compositional differences. Each dot is a sample, with colors and point shapes corresponding to the sample condition. **I** UMAP embedding of joint alignment of the Benign bone marrow stromal cells, color coded by the cell type. **J** Heatmap of scaled normalized expression for representative marker gene expression in stromal cell populations

To determine cell type signature genes, non-parametric Wilcoxon rank sum test was performed to find DEGs (differential expressed genes) among clusters using getPerCellTypeDE function in Conos [14]. DEGs were ranked by *p*-value determined *Z* score and filtered by *Z* score of more than 3. Major cell populations and cell subtypes were annotated using well-established marker genes. The detailed gene list can be found in Additional file 1: Table S4. Bone marrow and ccRCC primary tissue are annotated separately. We then verify the major cell annotations through joint integration (Fig. 1F). We first integrate all BM samples, including *Healthy*, *Involved*, *Distal*, and *Bone Met* samples. In total, 24 major clusters were obtained. To further confirm the cell annotations, we collected human bone marrow scRNA-seq data from HCA and Oetjen et al. [10, 16] and integrate our data with public datasets and perform single-cell reference mapping.

### Differential gene expression analysis

To analyze differential expressed genes between sample fractions for the same cell type (for example metastatic tumor cells vs. primary tumor cells), pseudo-bulk differential expression analysis [17] was applied by aggregating the counts of a group of cells from the same individual. We used estimateDEPerCellType function in Cacoa (v0.4.0) [18] to conduct pseudo-bulk differential expression analysis. After identification of the cell type identities of the scRNA-seq clusters, we aggregated the counts to the sample level for each cell type, and then used DESeq2 with the Wald test and the parameter independentFiltering = TRUE for differential expression analysis. A minimal number of 10 cells (of the selected cell type) and maximum 320 cells (average tumor cells per sample; down sampling if exceed) were required for a sample to be included in the comparison. To control for the variation in samples, we performed leave-one-out resampling procedure on samples and repeat this process 100 times (resampling.method = ‘loo’, n.resamplings = 100, min.cell.count = 10, n.cells.subsample = 320). Significant differential expressed genes (DEG) were defined as those with adjusted *p* values below 0.05 and log fold changes exceeding 1.5 (Fig. 5E).

### Cluster-based cell composition analysis

For cluster-based cell proportion analysis, we measure the relative cell proportion differences within the major cell population. In this analysis, we required at least 50 cells for the major cell population and measure the fraction of cell types per sample to avoid skewing the findings by a few individual samples. Statistical significance of proportion differences was evaluated using two-sided Wilcoxon rank sum test, followed by BH multiple testing correction.

### Cluster-free cell composition analysis

We use cell density to estimate cluster-free compositional changes by cacoa (v0.4.0) [18] pacakge. Briefly, UMAP embedding space is split into a grid of 400 × 400 bins, and 2D kernel density was estimated on UMAP. To account for the varying number of cells per sample, 2D kernel density was estimated for each individual sample. Then, average cell density per sample condition is shown as Fig. 3B. Then, the difference of sample densities between conditions is estimated for each data point (by default, using Wilcoxon test statistics).

### Estimate expression distance

Expression differences between matching subpopulations were calculated using estimateExpressionShiftMagnitudes function from cacoa (v0.4.0) [18]. Briefly, we first define “pseudo-bulk” RNA-seq measurements for each subpopulation in each sample and then calculate correlation distances between all pairs of samples. The overall expression distance is determined as a normalized weighted sum of correlation distances across all cell subpopulations contained in both samples, with the weight equal to the subpopulation proportion. Expression distances between samples are further projected to 2D space using multidimensional scaling (MSD) method with plotExpressionDistanceEmbedding function.

### Gene Ontology and Gene Set Enrichment Analysis

We use Cacoa [18] to perform Gene Ontology and Gene Set Enrichment Analysis. Cacoa uses clusterProfiler (v4.6.0) [19] functions for Gene Ontology (GO) and Gene Set Enrichment Analysis (GSEA). In all cases, Cacoa define gene universe as the set of all genes, expressed in at least 5% of cells of the analyzed cell type. The visualization function “dotplot” provided by clusterProfiler was used to generate the GO enrichment plots.

### Gene set signature score

We used a gene set signature score to measure cell states in different cell subsets and conditions. The signature scores were calculated as average expression values of the genes in a given set. Specifically, we first calculated the signature score for each cell as an average normalized (for cell size) gene expression magnitudes, and then the signature score for each sample was computed as the mean across all cells. All signature gene modules are listed in the Additional file 1: Table S5. The statistical significance was assessed using the two-sided Wilcoxon rank-sum test. Furthermore, we perform Benjamini-Hochberg (BH) multiple testing correction to ensure robustness of the results (*p < 0.05, **p < 0.01, ***p < 0.001).

### Ligand receptor analysis

To delineate the ligand-receptor (LR) interaction pair in ccRCC Bone Met single-cell data, we download LR pairs from CellPhoneDB (v3.1) [20] as background and use a similar approach described in CellPhoneDB to test if LR expression is significantly higher in certain cell types than it would be from a random cell type pairing. We first calculate ligand and receptor gene expression ratio scores for each cell type, requiring the genes that are at least expressed in 10% of cells within that cell type. To obtain the signal strength of a LR-pair in two corresponding cell types, we rely on the joint expression distribution of the associated genes. Specifically, we compute the LR-pair score given a cell type A and cell type B as the product of average expression of the ligand from cell type A and receptor for cell type B. We observe such a product might lead to an inflation of LR pairs that are in actuality not present in the environment. To filter out the statistically significant (*p* value 0.05) interactions, we further randomly shuffle the cluster labels of all cell types and re-calculate LR-pair score across 1000 permutations. This background is used as null distribution to evaluate the *p*-value for the target LR-pair interaction. In addition, we also evaluated ligand and receptor expression, requiring both ligand and receptor highly expressed in corresponding cell type. The getDifferentialGenes function from Conos [14] was used to derive DEG from each cell type and genes. We next screened each of the LR pair using *p*-value determined ligand *Z* score > 4 and receptor *Z* score > 0. The detailed LR list can be found in Additional file 1: Table S6.

### inferCNV analysis

To identify the copy-number variations of tumor cells from normal epithelial cells, we used interCNV (v1.3.3) [21] for inferring large-scale chromosomal copy-number variations. As ccRCC malignant cells originate from proximal tube epithelial cells, we performed inferCNV on tumor cells using the proximal tube cells as the reference “normal” cells.

### Survival analysis

To test if a given signature predicts survival, we first computed the average expression of the signature in each tumor based on the bulk RNA-Seq data. Next, we stratified the patients into two groups according to the average expression of the signature: high or low expression correspond to the top or bottom 25% of the population. We used a two-sided log-rank test to examine if there was a significant difference between patient groups in terms of their survival. R package survival (v3.5.0) and survminer (v0.4.9) were used to draw Kaplan Meier (KM) plot. In addition, we use Cox regression to analyze the potential technical factors that associated with patient survival. We included age and disease stage into the Cox regression model. Given the presence of age and disease stage, Macro-2 and MSC-2 signature still show a significant relationship with patient OS and PFS survival (Additional file 1: Table S7). In order to assess the stability of the list of signature genes, we performed a bootstrap resampling. This involved randomly selecting subsets of the signature genes and repeating the analysis 200 times. We then calculated *p*-values for each round of resampling and determined the statistical significance by reporting the 0.90th quantile of the sampled *p*-values.

### Flow cytometry analysis for myeloid and T cells

Samples from patients with RCC bone metastases were used for FACS analysis. Cells from human Bone Met and Distal BM samples were surface stained with a lymphoid antibody panel (Additional file 1: Table S8). Cells were washed once with 2% FBS-PBS (v/v). For intracellular staining to detect Treg infiltration, cells were fixed and permeabilized with Cytofix/Cytoperm (BD Biosciences, San Jose, CA) for 20 min at 4 °C, followed by one wash with 1 × Perm/Wash buffer (BD Biosciences, San Jose, CA). Cells were incubated overnight at 4 °C with anti-FoxP3-AF488, washed once in Perm/Wash buffer, and finally resuspended in Perm/Wash buffer for analysis. We acquired cell fluorescence data using a BD FACSAria II flow cytometer and used FlowJo (BD Biosciences, San Jose, CA) for analysis.

### Reverse transcription-quantitative PCR (RT-qPCR)

Total RNA from snap-frozen tissues or sorted cells was extracted using Direct-zol RNA MiniPrep Kit (Zymo Research, R2052) or RNeasy Micro Kit (Qiagen, 74,004). cDNA was synthesized from total RNA using iScript cDNA Synthesis Kit (Bio-Rad, 1,708,891). qPCR was performed using iTaq Universal SYBR Green Supermix (Bio-Rad, 1,725,121) on a CFX384 Real-Time System (Bio-Rad). The data were analyzed using the 2-ΔΔCt method. ACTB was used as housekeeping genes. The following primers were used for qPCR analysis: ACTB, AGAGCTACGAGCTGCCTGAC, AGCACTGTGTTGGCGTACAG; FN1, ACAACACCGAGGTGACTGAGAC, GGACACAACGATGCTTCCTGAG; FAP, GGAAGTGCCTGTTCCAGCAATG, TGTCTGCCAGTCTTCCCTGAAG; CCL18, GTTGACTATTCTGAAACCAGCCC, GTCGCTGATGTATTTCTGGACCC.

### Multiplex immunofluorescence

Multiplex immunofluorescence staining was performed using PANO 4-plex IHC kit (cat 10,001,100,100, Panovue). We performed the fluorescent dyes by using the Mouse anti Human *CD90* antibody, clone F15-42–1 (Dako, MA5-16,671), and *RANKL* rabbit anti-human antibody (PA5-110,268). After applying different primary antibodies, horseradish peroxidase-conjugated secondary antibody incubation and tyramide signal amplification were conducted. Following this, the slides were microwaved heat-treated. After labeling all human antigens, DAPI (SIGMA-ALDRICH, D9542) was used to stain the nuclei. Fluorescent images were captured by Confocal microscopes Leica SPE (Leica).

### Statistical analysis

*P* values < 0.05 were considered significant. Two-sided Wilcoxon rank sum test was used to assess significance in bulk seq and scRNA-seq analyses unless otherwise stated.

## Results

### The landscape of immune and stromal cells within human ccRCC bone metastasis

To define the microenvironment of ccRCC bone metastasis, we performed scRNA-seq on fresh patient samples. Our samples included metastatic tissue (*Bone Met*) and liquid BM at the vertebral level of spinal cord compression (*Involved*) as well as liquid BM from a different vertebral body distant from the tumor site but within the surgical field (*Distal*). In addition, we included bone and bone marrow stroma from patients undergoing hip replacement surgery and incorporated publicly available BM single-cell data from healthy donor controls (*Healthy*) and ccRCC primary tumors (cc*RCC Primary*) from our previous study [10, 11] (Fig. 1A). Among those data, we have two special patients (BM1 and BM2), where we collected the primary tumor, adjacent-normal kidney tissue, metastatic tumor, and involved and distal bone marrows from the same patient at diagnosis. All patients had a historic diagnosis of ccRCC and had standard pathologic evaluation to confirm ccRCC in the bone marrow within tissue sampled at the time of spinal decompression surgery (Fig. 1B and Additional file 2: Fig. S1A). Detailed clinical and pathological information, including tumor stage and treatment information, are available in Additional file 1: Table S1. Following quality control, including doublet removal and mitochondrial genes filtering, 264,681 cells were obtained. Conos [14] was used to integrate the multiple samples separately for primary and metastatic tumors. Unsupervised clustering of BM samples revealed 24 clusters including immune cells: T cells, natural killer (NK) cells, myeloid cells; stromal cells: MSCs, endothelial cells, pericytes (Fig. 1C–E and Additional file 2: Fig. S1B). To ensure the robust of scRNA-seq data integration, we re-analyzed the data using Seurat [15] pipeline, which shows consistent embedding and clustering (Additional file 2: Fig. S1C). Integrating primary and metastatic tumors confirms the cell identity and reveals cellular composition shifts in myeloid cell subsets, MSCs and tumor cells (Fig. 1F and Additional file 2: Fig. S1D). ccRCC tumor cells arise from epithelial cells of the proximal convoluted tubules, which do not exist in the normal BM [22, 23]. We could therefore distinguish malignant cells by their epithelial origin, which differs from the immune and stromal cells. We identified 1941 malignant cells by their expression of a panel of markers including KRT8, KRT18, and CA9 [24] (Additional file 2: Fig. S1E). Tumor cell identity was validated by inferred copy number aberrations (CNVs), showing notable inter-patient variation (Additional file 2: Fig. S1F).

Focusing on bone marrows, cellular composition analysis between samples revealed cell shifts in multiple lineages. The largest increase was observed in macrophages, regulatory T cells (Tregs), and CTLs in the *Bone Met* fraction, while naïve T cells and T helper cells were significantly decreased (Fig. 1G and Additional file 2: Fig. S1G). Complementary to the shifts of cell abundance between malignant and non-malignant BM, we examined transcriptional state differences using a weighted expression distance measurement [18]. There was significantly more variability between the samples collected from patient metastases as compared to control samples, suggesting broader complexity and heterogeneity of the bone metastatic microenvironment (Additional file 2: Fig. S1H). Expression distances between samples were projected in 2-dimensions using multidimensional scaling (Fig. 1H), to illustrate that the overall similarity of cell state in the different sample fractions consistently separates metastatic and non-metastatic BM.

Interestingly, stromal cell populations were readily detected in the *Bone Met* fraction, which contrasts to our previous study in prostate cancer bone metastases where there was a paucity of stromal cells [7]. We analyzed the transcriptome of enriched stromal cells from non-malignant BM (Fig. 1A). *Benign* BM revealed 6 subclusters including endothelial cells (VWF, PLVAP, CD34, CLDN5), osteoblasts (BGLAP, RUNX2, SPP1, NCAM1), and two pericytes clusters (Pericyte1: MYH11, DSTN, ACTA2; Pericyte2: MYL6, STEAP4, SEPT7, MYO1B) (Fig. 1I–J). MSCs expressing LEPR [25] were found in high abundance in the benign bone marrow (MSC1: CXCL12, LEPR, VCAN, SEPP1, VCAM1). In addition, a smaller fibroblast population was identified with high expression of APOD, MFAP4, IGFBP6, MGP) (Fig. 1J).

### RCC bone metastases exhibit increased recruitment of a distinct tumor-associated macrophage subpopulation

**Fig. 2:**
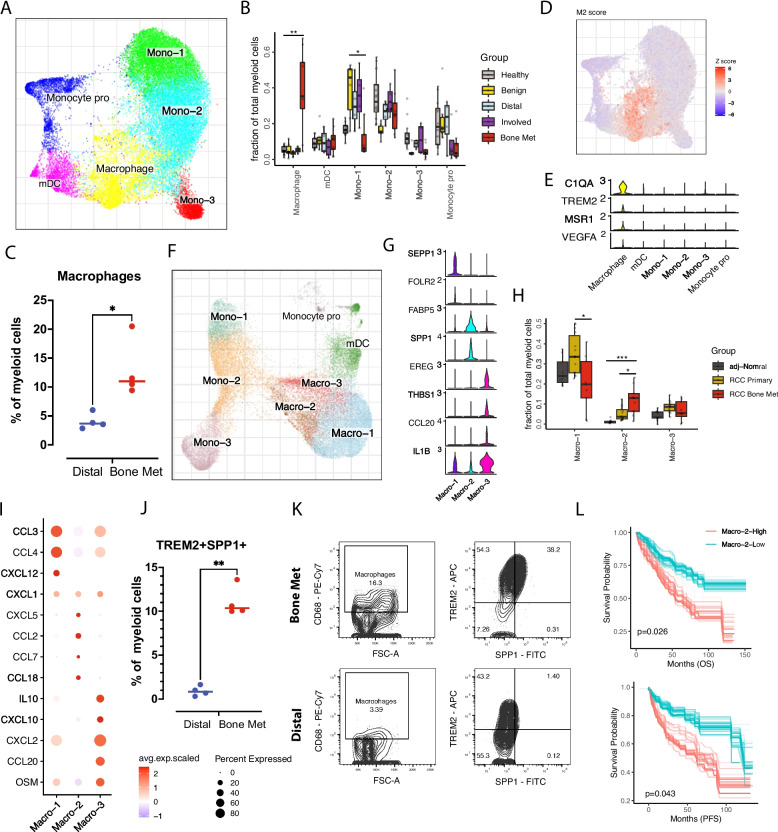
Distinct tumor-associated macrophage subpopulations in ccRCC bone metastasis. **A** UMAP joint embedding showing myeloid cell subsets. **B** Comparison of relative cell abundance of myeloid cell subsets between *Bone Met* (*n* = 9) and different control fractions (*Healthy n* = 12, *Benign n* = 7, *Involved n* = 4, *Distal n* = 4). Statistics are accessed with two-sided Wilcoxon rank sum test and BH multiple testing correction. (*p < 0.05, ***p < 0.001, Additional file 1: Table S3). **C** Box plot showing the percent of Macrophages (CD68 +) of the CD45 + / CD11b + population in *Bone Met* (*n* = 4) and *Distal* (*n* = 4) by flow cytometry. Statistical significance determined using two-sided *t*-test (*p < 0.05). **D** Scaled average expression of M2 signature genes visualized on UMAP embedding. **E** Representative M2 marker gene expression shown on violin plot. **F** UMAP joint embedding showing integrated analysis of myeloid cells from ccRCC primary tumor and bone metastasis tumor. **G** Violin plot showing representative marker gene expression across three macrophage subpopulations. **H** Box plot comparing proportion of macrophage populations across bone metastatic ccRCC (*n* = 9), primary ccRCC (*n* = 14), and adjacent normal tissue (*n* = 9). Statistics are accessed with two-sided Wilcoxon rank sum test and BH multiple testing correction. (*p < 0.05, ***p < 0.001). **I** Dot plots showing cytokine gene expression across different macrophage subsets. The color represents scaled average expression of marker genes in each cell type, and the size indicates the proportion of cells expressing marker genes. **J**, **K** Gating strategy for enrichment of TREM2 + SPP1 + macrophages. Labels above the flow plots refer to the parent population in the percentages are of the parent gate (**J**). Box plot showing the percent of TREM2 + /SPP1 + cells for the CD45 + / CD11b + population in *Bone Met* (*n* = 4) and *Distal* (*n* = 4). Statistical significance was determined using two-sided *t*-test (K). **L** Kaplan–Meier curves showing ccRCC samples with higher Macro-2 signature gene (SPP1, FABP5, CCL18, CXCL5, CCL7) expression have worse overall survival (top; *n* = 533) and progression-free survival (bottom; *n* = 435) in TCGA KIRC data. Patients were stratified into two groups based on the average expression (binary: top 25% versus bottom 25%) of Macro-2 signatures. *p* value was evaluated using Log-rank test. Bootstrap resampling was performed on signature genes and *p*-value was calculated using the 95% reproducibility power *p*-value (see the “Methods” section)

The bone marrow contains abundant immune cells, including different myeloid and T cell lineages [10]. Myeloid cells play an instrumental role in the TME and contribute to both tumorigenesis and metastasis [26, 27]. Within the ccRCC BM microenvironment, we identified 6 myeloid subclusters (Fig. 2A and Additional file 2: Fig. S2A): classical monocytes (Mono-1/Mono-2: S100A8, S100A9, and CD14), non-classical monocytes (Mono-3: lacked CD14 expression but expressed FCGR3A (CD16)), monocyte progenitor cells (expressed high levels of MPO and MKI67), dendritic cells (DCs, expressed CD1C and FCER1A) (Additional file 2: Fig. S2B,C). We also identified a tumor-associated macrophage (TAM) population that was specifically enriched in the patient *Bone Met* fraction (Fig. 2B and Additional file 2: Fig. S2A, D). Flow cytometry analysis confirmed a higher infiltration of macrophages in *Bone Met* tissues compared to *Distal* BM tissue (Fig. 2C). TAMs were marked by the high expression of C1QA, C1QB, and CD163 [7] and displayed an M2-like phenotype, with high levels of IL10, MSR1, CD163, and TREM2 [28–30] (Fig. 2D, E and Additional file 2: Fig. S2B, E). TREM2 + macrophages have been identified in advanced ccRCC patients and are associated with T cell exhaustion and anti-PD-1 resistance [31]. IL10 can mediate immune suppressive effects by directly suppressing lymphocyte responses and indirectly blocking DC functions. The finding of M2-like TAMs within ccRCC bone metastasis suggests an immunosuppressive microenvironment favoring tumor cell growth. Macrophages are heterogeneous and several populations of macrophages have been described in primary ccRCC [32]. To define the subpopulations of macrophages and the association with TAMs identified in primary ccRCC, we performed integrated analysis of myeloid cells from primary ccRCC patients. Myeloid sub-clustering revealed three distinct macrophage populations (Macro1-3) (Fig. 2F–H). Macro-1 was defined by expression of SEPP1, PDK4 and FOLR2, Macro-2 by expression of FABP5, VIM and SPP1, and Macro-3 by expression of CCL20, EREG, THBS1, and IL1B (Fig. 2G and Additional file 2: Fig. S2F).

The Macro-1 cluster was transcriptionally similar to tissue-resident macrophages [33] as reported in primary ccRCC, breast and lung cancers [34], and expressed markers such as SEPP1, FOLR2, CCL3, CCL4, and CXCL12. Macro-2 showed high expression of SPP1, CXCL5, CCL2, CCL7, and CCL18 (Fig. 2I and Additional file 2: Fig. S2F). Compared to samples from primary ccRCC, the composition of macrophages in bone metastases demonstrated a shift towards an increased fraction of Macro-2 (Fig. 2H). SPP1 is involved in bone formation and in anchoring of osteoclasts to the bone remodeling matrix [35]. CCL18 is reported to promote metastasis in breast cancer, colon cancer, and squamous cell carcinoma [36, 37]. CXCL5 is elevated in tumor tissues and is positively associated with lymphatic metastasis and tumor differentiation [38, 39]. Flow cytometric analysis of freshly cryopreserved samples validated the increase of TREM2 + SPP1 + macrophages in *Bone Met* compared to *Distal* BM tissue (Fig. 2J, K). To evaluate the potential prognostic value of different macrophage subpopulations, we ran survival analysis on public ccRCC bulk RNA sequencing data based on key marker gene expression from our scRNA-seq dataset. Interestingly, Macro-2 was associated with poor progression-free and overall survival (Fig. 2L). Furthermore, differential gene expression analysis comparing Macro-2 from bone metastases versus primary ccRCC showed that lymphocyte and T cell activation genes are downregulated (Additional file 2: Fig. S2G), suggestive of a immunosuppression within the metastatic TME. Collectively, our data revealed distinct tumor-associated macrophage subpopulations in the metastatic TME and suggested the potential role of Macro-2 in tumor bone metastases.

### Sustained T cells dysfunction in ccRCC primary and bone metastatic tumors

**Fig. 3:**
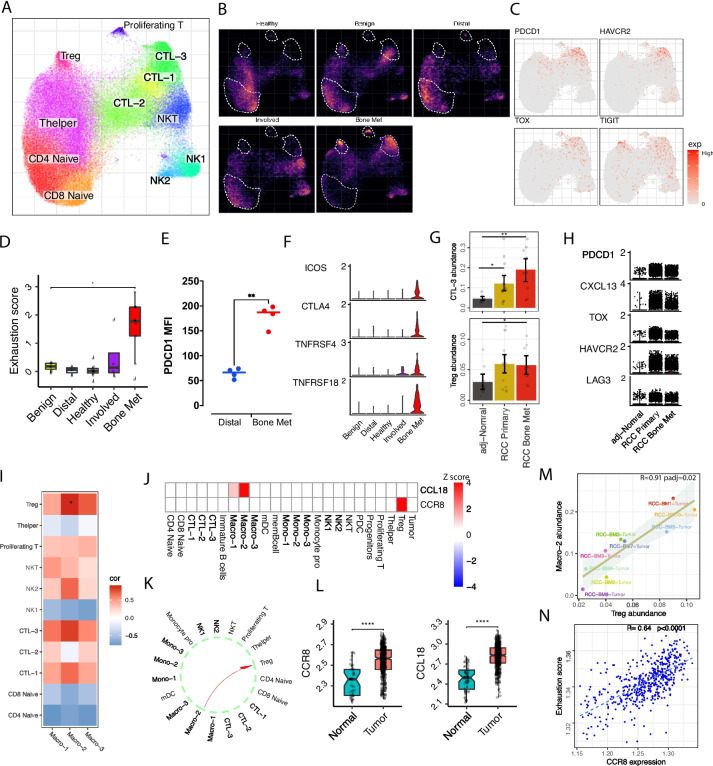
Dysfunctional T cells correlate with Macro-2. **A** UMAP embedding demonstrating T cell subpopulations. **B** Visualization of the average cell density across *Bone Met* (*n* = 9) and multiple control conditions (*Healthy n* = 12, *Benign n* = 7, *Involved n* = 4, *Distal n* = 4), using embedding density estimates. Brighter colors correspond to denser regions (see the “Methods” section). **C** Expression of representative T cell exhaustion markers on UMAP embedding. **D** Box plots showing T cell exhaustion score within CTL-3 across *Bone Met* (*n* = 9) and control conditions (*Healthy n* = 12, *Benign n* = 7, *Involved n* = 4, *Distal n* = 4). Statistics are accessed with two-sided Wilcoxon rank sum test and BH multiple testing correction (*p < 0.05). For box plots, center line represents the median and box limits represent upper and lower quartiles, and whiskers depict 1.5 × the interquartile range (IQR). **E** Comparison of PDCD1 expression (MFI) in *Distal* (*n* = 4) and *Bone Met* (*n* = 4) samples. Statistical significance determined using two-sided *t*-test (*p < 0.05). **F** ICOS, CTLA4, TNFRSF4, and TNFRSF18 expression in Tregs shown as violin plot. **G** Bar plot showing CTL-3 (top) and Treg abundance (bottom) comparing *RCC Bone Met* (*n* = 9) with *RCC Primary* (*n* = 14) and *adjacent normal* (*n* = 9) fractions. Statistics are accessed with two-sided Wilcoxon rank sum test (*p < 0.05, **p < 0.01). **H** Violin plot showing representative exhausted T cell signature gene expression in CTL-3 comparing *RCC Bone Met* with *RCC Primary* and *adjacent normal* fractions. **I** Correlations of the cell abundance between myeloid and T cell subsets shown as heatmap. Significance was assessed using Pearson correlation test and BH multiple testing correction. Color represents correlation coefficient and star presents the significance. (*p < 0.05). **J** Heatmap showing scaled average expression of CCL18 and CCR8 in major cell populations. **K** Circle plots showing the inferred CCL18-CCR8 signaling between Macro-2 and Treg. **L** Box plot showing CCL18 and CCR8 abundance in tumor (*n* = 72) compared to adjacent normal (*n* = 533) tissue in TCGA KIRC. Statistics are accessed with two-sided Wilcoxon rank sum test (****p < 0.0001). **M** Correlation of CCR8 expression in Tregs and CTL-3 exhaustion score in CTL-3 is shown as a scatter plot. Pearson linear correlation estimate, and *p*-values are shown. The error band indicates 95% confidence interval. **N** Correlation of CCR8 expression and CTL-3 exhaustion score is shown as a scatter plot for TCGA KIRC data (*n* = 533). Pearson linear correlation estimate, and *p*-values are shown

T cells play a central role in the anti-tumor immune response [40]. Dysfunctional or exhausted CD8 + cytotoxic T cells have been identified in ccRCC and the metastatic TME [32, 41] We found clusters of naïve CD4 (SELL, CCR7, CD4), naïve CD8 (SELL, CCR7, CD8A), T helper (CD4, RORC), Treg (FOXP3, CTLA4, IL2RA), NK1 (GZMB, FGFBP2, NKG7, KLRD1), NK2 (XCL1, XCL2, CMC1), proliferating T cells (TOP2A, MKI67), and three subtypes of CTLs, CTL-1 (CD8A, KLRG1, CMC1), CTL-2 (KLRB1, GZMK, IL7R), and CTL-3 (PDCD1, HAVCR2, IFNG, GZMK) (Fig. 3A, B and Additional file 2: Fig. S3A-C). The CTL-3 cluster exhibited high expression of exhaustion signature genes (HAVCR2, PDCD1, TOX, TIGIT) (Fig. 3C). The dysfunctional cell state in CTL-3 was compared across different sample fractions by exhaustion signature score analysis, demonstrating the highest exhaustion in the *Bone Met* compartment (Fig. 3D). Moreover, cell composition analysis demonstrated a significant increase of CTL-3, Tregs, and the proliferating T cells in the *Bone Met* fraction, whereas naïve CD4 and naïve CD8 were reduced (Fig. 3B and Additional file 2: Fig. S3D, E). Flow cytometric analysis of freshly cryopreserved samples further validated the upregulation of PDCD1 and increased PDCD1 + CD8 + cells in the *Bone Met* compartment (Fig. 3E, and Additional file 2: Fig. S3F,G). Together, this suggests that CTLs have lost their immune responsive capacity in the metastatic BM TME. Tregs are critical to the maintenance of immune tolerance and suppression [42] Compared to non-malignant BM TME, we observed increased expression of Treg costimulatory molecules (TNFRSF4, TNFRSF18, ICOS) and the inhibitory molecule CTLA4, pointing to an immune suppression and T cell escape within the *Bone Met* location [43, 44] by Tregs (Fig. 3F). We further compared ccRCC *Bone Met* T cell compartments with *ccRCC primary* tumors (Additional file 2: Fig. S3H). The two immune suppressive components (CTL-3 and Tregs) significantly increased in primary tumors and were sustained in metastatic lesion (Fig. 3G). Examination of CTL exhaustion and Treg costimulatory molecules expression further verified dysfunctional T cell states both in RCC primary and bone metastatic tumors (Fig. 3H and Additional file 2: Fig. S3I, J).

### Dysfunctional T cells correlate with the myeloid TAM-2 population

Our data suggest that T cells and TAMs may cooperate to contribute to an immunosuppressive TME. The Macro-2 (TAM-2) abundance was significantly correlated with Treg abundance and CTL-3 abundance (Fig. 3I and Additional file 2: Fig. S4A), pointing towards an interplay that might favor immune suppression [45]. Ligand-receptor analysis identified biologically important interactions between Macro-2 and CTL-3/Treg populations, including known immune suppressive interactions such as CD86/CTLA4 [46] and IL10/IL10RB [47] (Additional file 2: Fig. S4B). Interestingly, the expression of CCL18 was specific to the Macro-2 population, and receptor CCR8 was exclusive to Tregs, suggesting a biologically relevant interaction (Fig. 3J, K). We validated this by using FACS to sort TREM2 + SPP1 + macrophages and confirmed the expression of CCL18 with RT-qPCR in multiple patient samples (Additional file 2: Fig. S4C). Compared to *Distal* BM, TREM2 + SPP1 + macrophages significantly increased in *Bone Met* tissue (Fig. 2K). As limited TREM2 + SPP1 + macrophages were obtained in *Distal* BM, we merge all *Distal* TREM2 + SPP1 + macrophages together for as control (#1). Additionally, we use FACS to demonstrate CCR8 protein expression in Tregs, as CCR8 is a cell surface protein (Additional file 2: Fig. S4D). Moreover, analysis of bulk RNA-seq data shows that both CCL18 and CCR8 are significantly upregulated in tumors (Fig. 3L). CCL18 expressed from TAMs plays a critical role in immune and inflammation responses, and its receptor CCR8 marks suppressive Treg cells within the tumor [48] suggesting the immunosuppressive potential of the CCL18-CCR8 axis in bone metastatic ccRCC. To further investigate the immunosuppressive properties of CCL18-CCR8, we perform correlation analysis between CCR8 expression from Tregs and exhaustion signature score from CTL-3. Interestingly, a significant correlation coefficient was observed and was further confirmed in bulk RNA-seq data. (Fig. 3M, N). Taken together, our data show that bone marrow in metastatic patients is immune-suppressive, featuring increased TAMs, exhausted CD8 + T cells, and Tregs, indicating the potential interactions among immune suppressive components (Additional file 2: Fig. S4E).

### A tumor-specific mesenchymal stromal cell population is associated with worse patient survival

**Fig. 4:**
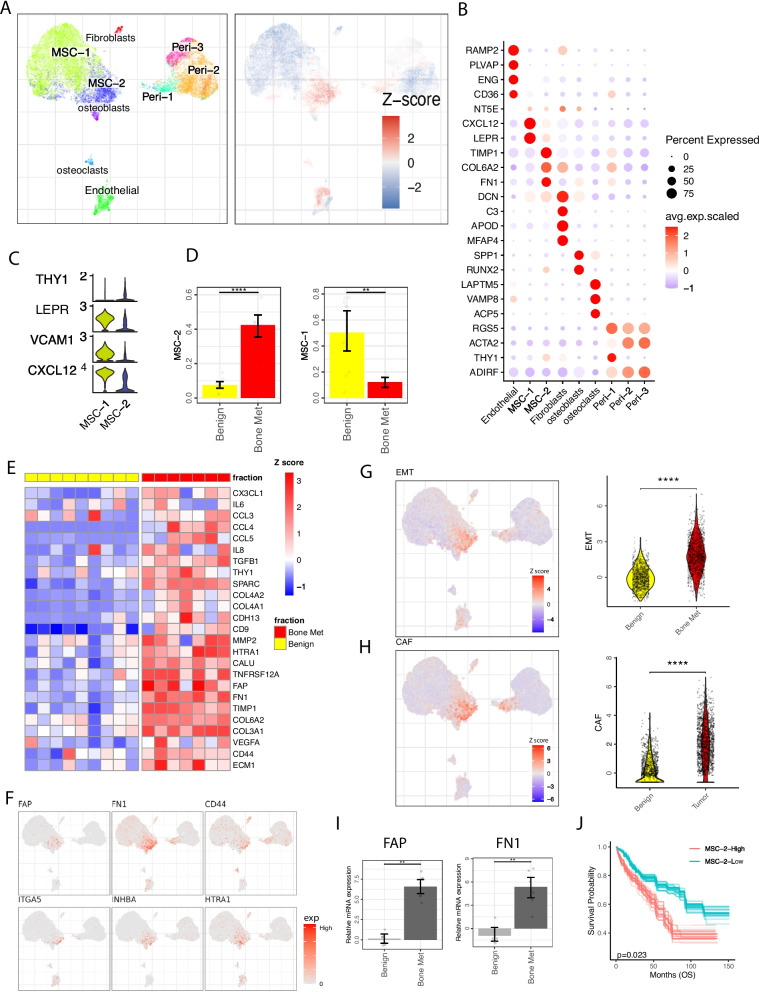
A distinct tumor-associated mesenchymal stroma cell (MSC) in ccRCC bone metastasis displaying CAFs phenotype. **A** UMAP embedding showing stromal cell subpopulations (left) and cell density difference comparing tumor with benign condition (right). *Z* score evaluates whether the cells are enriched in tumor (high *Z* score, red) or benign (low *Z* score, blue) condition. **B** Dot plot representing key-marker gene expression of stromal cell types. The color represents scaled average expression of marker genes in each cell type, and the size indicates the proportion of cells expressing marker genes. **C** Visualization of MSC marker gene expression shown as violin plot. **D** Bar plot illustrates cell abundance differences between *Bone Met* (*n* = 9) and *Benign* (*n* = 9) conditions for MSC-1 (left) and MSC-2 (right). Significance was assessed using two-sided Wilcoxon rank sum test. **E** Heatmap showing scaled average gene expression in MSC-2 across *Bone Met* and *Benign* conditions for each patient (column). **F** UMAP visualization of representative EMT and CAFs signature gene expression in stromal cells. **G** EMT gene signature score in stromal cells, UMAP visualization of EMT score (left). Violin plots of the EMT gene signature score in *Bone Met* and *Benign* MSC-2 cells (right). Significance was assessed using two-sided Wilcoxon rank sum test (****p < 0.0001). **H** Similar to Fig. 4G, showing CAF gene signature score (****p < 0.0001). **I** Bar plot showing relative mRNA expression (log fold change) of FAP and FN1 in *Benign* (*n* = 5) and *Bone Met* (*n* = 7) tissue by RT-qPCR. Data are expressed using the 2 − ∆∆Ct method. Gene expression levels were normalized to the benign control. Statistical significance determined using two-sided *t*-test. **J** Kaplan–Meier curves showing ccRCC samples with higher MSC-2 signature gene (COL6A2, FN1, TIMP1, COL3A1, COL1A2) expression have worse progression-free and overall survival (*n* = 533) in TCGA KIRC data. Patients were stratified into two groups based on the average expression (binary: top 25% versus bottom 25%) of MSC-2 signatures. *p* value was evaluated using Log-rank test. Bootstrap resampling was performed on signature genes and *p*-value was calculated using the 95% reproducibility power *p*-value (see the “Methods” section). For box plots, center line represents the median and box limits represent upper and lower quartiles, and whiskers depict 1.5 × the interquartile range (IQR)

Stromal cells of normal human BM and bone metastatic tumors have not yet been characterized at the single-cell level. We identified osteoclasts (VAMP8, CAP5) [49] osteoblasts (SPP1, RUNX2) [50], fibroblasts (DCN, APOD, MFAP4) [51], endothelial cells (PLVAP, RAMP2) [52], two MSC (NT5E, CXCL12, LEPR), and three pericyte (RGS5, ACTA2) subpopulations [53] (Fig. 4A, B and Additional file 2: Fig. S5A, B). Comparing the distribution of stromal cells demonstrated changes in cell composition of MSCs, endothelial cells, and pericytes within the metastatic *Bone Met* fraction (Fig. 4A and Additional file 2: Fig. S5C-E), suggesting the tumor-induced stroma remodeling.

The MSC subsets were characterized by the expression of key MSC markers LEPR, NT5E, THY1 (CD90), VCAM1, and the known hematopoietic stem cell niche factor CXCL12 [25] (Fig. 4B). MSC-2 cluster maintained the expression of classic MSC markers NT5E, THY1(CD90) but had reduced expression of VCAM1, LEPR, and CXCL12 compared to MSC-1 (Fig. 4B, C and Additional file 2: Fig. S5A). The similar downregulation of CXCL12 expression was recently observed in bone marrow derived LEPR + MSCs in murine leukemia [25]. MSC-2 abundance was significantly increased in the *Bone Met* compartment compared to *Benign* (Fig. 4D) and displayed high expression level of EMT markers HTRA1, INHBA, and ITGA5 [54, 55] (Fig. 4E, F). Furthermore MSC-2 showed pronounced EMT signature score, particularly within *Bone Met* fraction (Fig. 4G). This elevated EMT score indicates a substantial degree of cell state plasticity and motility, which are recognized as key indicators of metastatic potential [56].

MSC-2 also demonstrated high expression of SPARC, a factor known to mediate the disruption of cell adhesion [57]. Multiple collagen-associated genes (COL6A2, COL3A1, COL4A1, COL4A2) were upregulated in the *Bone Met* MSC-2 cluster, indicative of active extracellular matrix remodeling, which was further supported by upregulated processes such of cell adhesion, tube morphogenesis, extracellular matrix organization, and collagen fibril organization [58] (Fig. 4E and Additional file 2: Fig. S5F). Additionally, MSC-2 shown high expression of cancer-associated fibroblast (CAFs) markers, including FAP, FN1, and CD44 [59] (Fig. 4F). The enhanced expression of FAP and FN1 were further validated using RT-qPCR in *Bone Met* samples (Fig. 4I). CAFs have been observed in multiple cancer types and are known to secrete factors (e.g., IL6, IL8, TGFB1) that can regulate cancer proliferation and metastasis [60]. Our analysis revealed that the CAF signature was predominantly found in the *Bone Met* MSC-2 cells and enriched in *Bone Met* fraction (Fig. 4E, H). Moreover, we generated gene signatures describing MSC-2 and restricted these signatures to MSC-2-specific genes (Methods). We utilize bulk RNA-seq data and found a significant upregulation of MSC-2 signature in tumor compared to adjacent normal tissue (Additional file 2: Fig. S5G). We then performed survival analysis separating bulk RNA-seq samples into MSC-2 high and MSC-2 low groups. MSC-2 signature was shown to be associated with poor progression-free and overall survival (Fig. 4J and Additional file 2: Fig. S5H). Our data provides evidence that CAFs phenotype of MSCs in the metastatic BM and are not shown in normal BM [61]. This observation implies a potential transition from MSC-1 to MSC-2 cells accompanied with a CAF-like and EMT-like transcriptional reprogramming in tumor bone metastasis cascade.

### EMT programs are enriched in metastatic ccRCC compared to primary ccRCC

The homing of the cancer cells to the bone marrow is a multi-step process that includes extravasation from the bloodstream, tissue invasion, disruption of normal bone marrow homeostasis, and ultimately the promotion of an immunosuppressive TME [62]. To better understand cell heterogeneity and the cellular programs that may drive tumor cell migration and metastasis, we also included comparison datasets of proximal tubule cells from adjacent normal kidney tissue (that are thought to be the origin of kidney cancer) and tumor cells from publicly available samples of primary ccRCC [11].

**Fig. 5:**
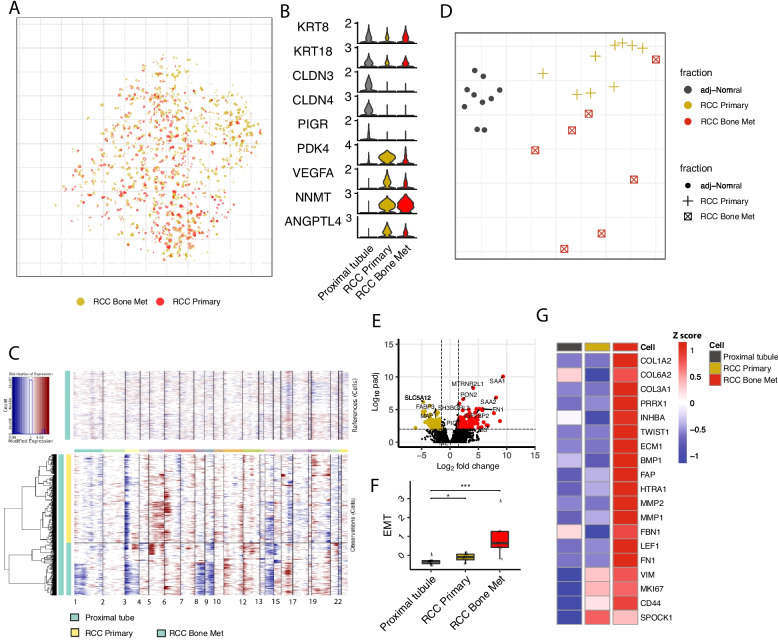
EMT programs promoting metastatic behavior are highly elevated in metastatic ccRCC. **A** Joint embedding of tumor cells from *ccRCC primary* and *ccRCC Bone Met* samples. **B** Violin plots of genes expressed in the proximal tubule of the *normal adjacent kidney* tissue, *ccRCC primary*, and *ccRCC Bone Met* tumor cells. **C** InferCNV analysis showing CNV pattern of metastatic and primary tumor cells taking proximal tube cells as control. **D** Pairwise expression distances between samples are shown using MDS embeddings. Each dot is a sample, with colors and point shapes corresponding to the sample condition. **E** Volcano plot illustrate differential expressed genes comparing bone metastatic tumor cells compared with primary ccRCC tumor cells. The vertical dashed lines show the cut-off for gene filtering (log2FoldChange 1.5 and − 1.5), and the horizontal dashed line signifies an adjusted *p* value of 0.01 (see the “Methods” section). **F** Box plot comparing the EMT gene signature score across proximal tubule of the *normal adjacent kidney* tissue, *ccRCC primary*, and *ccRCC Bone Met* tumor cells*.* For box plots, center line represents the median and box limits represent upper and lower quartiles, and whiskers depict 1.5 × the interquartile range (IQR). **G** Heatmap showing representative EMT signature genes expression in proximal tubule of the *normal adjacent kidney* tissue, *ccRCC primary*, and *ccRCC Bone Met* tumor cells. Color represents scaled average gene expression

High similarity was observed between malignant cell and proximal tube cells in joint alignment (Fig. 5A and Additional file 2: Fig. S6A), showing high expression of epithelial markers KRT8, KRT18 (Fig. 5B). However, the transcriptional profile changed in the tumor with a significant upregulation of ccRCC signature genes VEGFA, NDUFA4L2, and PDK4 [32] both in the primary and the metastatic setting (Fig. 5B and Additional file 2: Fig. S6B). Furthermore, we analyzed copy number variations (CNVs), taking proximal tube cells from adjacent normal kidney tissue as reference with inferCNV [21]. These inferred CNVs were consistent with previous reports of Chr3 loss in ccRCC patients and accumulated CNVs in metastatic ccRCC patients with additional loss of *Chr9* and *Chr14* [9] (Fig. 5C).

Patient-to-patient variability was most highly pronounced in the metastatic tumor cell fractions when compared to primary tumors or to normal epithelial proximal tubule cells (Additional file 2: Fig. S6C). This suggests a high degree of tumor cell transcriptional heterogeneity and may imply that the metastatic tumor has a higher degree of complexity and therefore might be more challenging to target. Further analysis of expression distances using multidimensional scaling resulted in consistent divergence of the transcriptional state of metastatic tumors (Fig. 5D). EMT programs have been widely considered to be drivers of tumor invasion and metastasis [56]. We examined the EMT program in primary and metastatic samples with a focus on the tumor cells and a comparison to normal proximal tubule cells. EMT signatures were significantly increased in the metastatic tumor cells (Fig. 5E, F) that is in agreement with previous report of EMT on tumor cells dissemination [6, 56]. Further differential gene expression analysis showed that tumor cells from bone metastases also differed from primary tumor cells with upregulation of actin cytoskeleton organization and extracellular matrix organization, key programs in EMT (Fig. 5G and Additional file 2: Fig. S6D).

### Tumor-associated MSCs trigger dysregulated bone remodeling within ccRCC metastasis

**Fig. 6:**
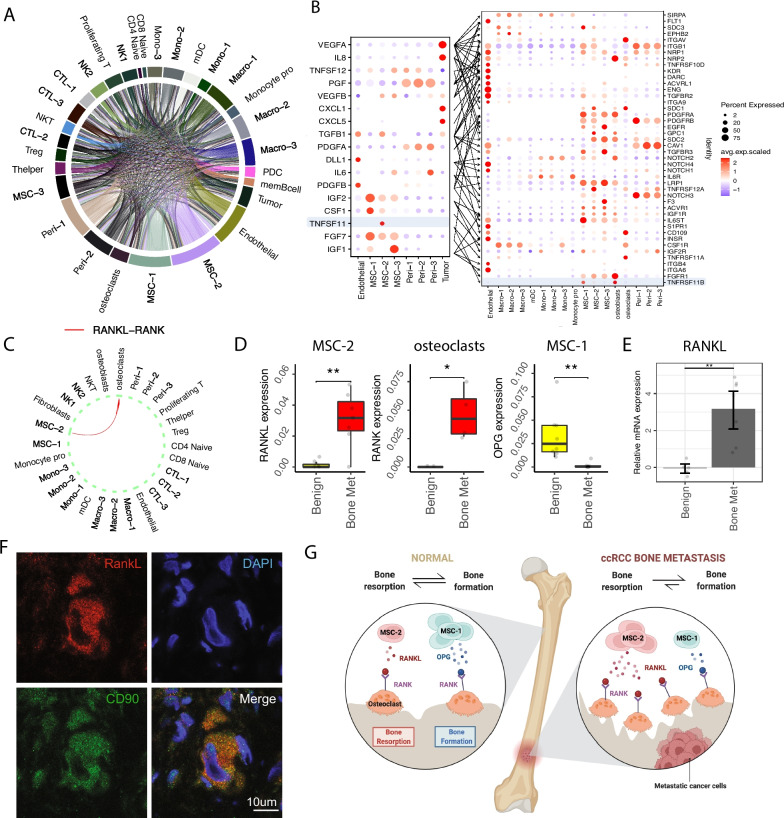
Tumor-associated MSCs source to bone remodeling of ccRCC bone metastasis. **A** Overview of number of significant ligand receptor pairs (Additional file 1: Table S6). **B** Bubble heatmap showing expression of ligand (left: tumor cells and stromal cell subsets) and receptor (right: stromal and myeloid subsets) pairs in different stromal and immune subsets. Dot size indicates expression ratio; colored represents average gene expression. **C** Circle plots showing the inferred RANKL-RANK signaling between MSC-2 and osteoclasts. **D** Box plot showing RANKL expression in MSC-2 and OPG expression in MSC-1 (*Benign n* = 8; *Bone Met n* = 7). Average gene expression was used, each dot represents a sample. Significance was assessed using two-sided Wilcoxon rank sum test (*p < 0.05, **p < 0.01). **E** Bar plot showing relative mRNA expression (log fold change) of RANKL in *Benign* (*n* = 5) and *Bone Met* (*n* = 7) tissue by RT-qPCR. Data are expressed using the 2 − ∆∆Ct method. Gene expression levels were normalized to the benign control. Statistical significance determined using two-sided *t*-test. **F** Immunostaining in tissue from bone metastatic ccRCC stained for RANKL, MSC-2 specific marker CD90, and DAPI. **G** Schematic illustration of MSC cell shift in mediating RANKL/OPG-RANK signaling pathway. For box plots, center line represents the median and box limits represent upper and lower quartiles, and whiskers depict 1.5 × the interquartile range (IQR)

Next, we focused on channels of communication between tumor cells and the TME that might explain the immune suppressive nature of the macrophages, the exhausted T cell populations, and the EMT changes in the tumor cells. We asked what channels might mediate growth and maintenance of the cancer in the BM? To answer this question, we performed a ligand-receptor analysis to identify cellular crosstalk among the different cell populations. In total, we identified 5317 channels as potential drivers of ccRCC bone metastases. While most of the channel interactions are within the different stromal cell subpopulations, we also identified significant interactions between stroma and immune cells (Fig. 6A and Additional file 2: Fig. S7A, B). With a focus on the interactions within stroma cells and myeloid subpopulations, we identified biologically important interactions involved in bone remodeling, including RANKL-RANK, Oncostatin M (OSM) and its receptor OSMR, and VEGF-KDR [63–65] (Fig. 6B, C and Additional file 2: Fig. S7B).

The RANKL/RANK/OPG signaling is critical in orchestrating osteoclasts maturation, bone modeling, and bone remodeling. We observed an increase of RANKL expression in *Bone Met* MSC-2 cells, along with an increase of the receptor RNAK expression in osteoclast (Fig. 6D, and Additional file 2: Fig. S7C). In addition, the decoy receptor OPG (acts as a RANK antagonist) was reduced in the *Bone Met* MSC-1 cells when compared to the *Benign* fraction (Fig. 6D). This observation suggests a specific activation of the RANKL-RANK axis within the tumor [66]. Tumor cells are believed to provide the source of RANKL production, and it has been demonstrated that RANKL-expressing tumor cells are attracted to the high local concentrations of RANK within the bone [5]. However, our analysis suggests that the source of RANKL is produced by the distinct tumor-associated MSC-2 population rather than tumor cells (Additional file 2: Fig. S7D). RT-qPCR validation confirmed the upregulation of RANKL in *Bone Met* samples compared to *Benign* control (Fig. 6E). Additionally, multiplex immunohistochemistry (mIHC), performed in situ on *Bone Met* tumor as the single-cell expression, confirmed the co-localization of CD90 and RANKL (Fig. 6F and Additional file 2: Fig. S7E). We further examined RANKL expression in two public scRNA-seq data from primary and advanced ccRCC patients [11, 41], showing an absence of RANKL expression in the tumor cells (Additional file 2: Fig. S7F). Therefore, we hypothesize that tumor-associated MSC-2 populations is the mediator of the bone remodeling observed in ccRCC bone metastasis patients through the channel of RANKL-RANK/OPG signaling. In line with this hypothesis, we observed changes in osteoblast and osteoclasts, the key regulators of bone formation and resorption, where dysregulation of bone remodeling is known to be involved in promoting metastases [4, 5]. RANK (receptor) expression is significantly enhanced in *Bone Met*-associated osteoclasts (Fig. 6D), and these osteoclasts displayed an upregulation of genes related to differentiation and activation [67, 68] (CA2, TCIRG1, CLCN7, OSTM1, and ANXA2), implying a program of active bone resorption. Meanwhile, osteoblasts showed reduced expression of genes related to osteoblast proliferation, mineralization, and connective tissue integrity (LRP5, ALPL, BGLAP and BMP4), indicative of impaired osteoblast-mediated bone formation [69] (Additional file 2: Fig. S7G). Taken together, our data suggested tumor-associated MSCs source to bone remodeling of ccRCC bone metastasis through dysregulation of the RANKL/OPG-RANK axis (Fig. 6G).

In addition to RANKL/OPG/RANK axis, OSM is of particular interest in the bone metastatic process because of its ability to independently stimulate the expression of RANKL. More specifically, OSM secreted by monocyte-derived macrophages can stimulate RANKL production through direct contact with MSCs via the OSM receptor [64]. Our results provide further support to the importance of this axis in metastatic ccRCC, as OSM expression was expressed in the Macro-1 and Macro-3 populations, while the expression of OSMR was found in tumor-associated MSC-2 cells (Additional file 2: Fig. S7B). In addition, tumor cells also showed increased expression of OSMR, which suggests the presence of the OSM-OSMR axis in metastatic ccRCC potentially acting as an independent RANKL-inducing pathway.

## Discussion

The tumor and immune microenvironment of primary and advanced ccRCC has been widely studied at the single-cell resolution [32, 41]. However, a deeper understanding of the cellular relationships within bone metastatic ccRCC has not been explored. Here, we used scRNA-seq to construct a single-cell transcriptomic atlas of the microenvironment of human ccRCC bone metastasis. Our analysis identified cells influencing ccRCC bone metastasis, including immunosuppressive TAMs and Tregs, and dysfunctional CTLs. We revealed an EMT cell state shift in a distinct MSC populations that promotes bone remodeling activity. Therapeutically modulating immune cells (e.g., immune checkpoint blockade) has been proven beneficial in ccRCC [41]. Targeting stromal cells in ccRCC bone metastases might as well be an effective therapeutic strategy.

Metastatic spread is often accompanied by tumor cell heterogeneity which may enable cancer cells to adapt to specific microenvironments and overcome metastatic barriers. Here, we observed significant inter-patient variability of malignant cells from metastatic patients. This suggests that distinct patterns of gene expression and mutational burden may be linked to different metastatic behaviors. Despite the variability of malignant cells in metastatic patients, we consistently observed an activated EMT program [70, 71].

TAMs are widely present in different TME. Removal or disruption of TAMs leads to reduced bone metastatic growth in breast and prostate cancer [7, 72]. We found a diversity of TAM subpopulations in metastatic sites. Among them, Macro-2 seems to be a key player in the tumor metastatic cascade, characterized by expression of SPP1, CCL18, CXCL5, CCL2, and CCL7. SPP1 + macrophages have been observed in lung adenocarcinoma lymph node metastasis and colon cancer liver metastasis [73, 74]. CXCL5 was reported to be involved in the formation of a premetastatic niche promoting breast cancer cells to proliferate and colonize in the bone [38, 39]. Cell–cell interaction analysis points to communication between Macro-2 and Tregs through CCL18-CCR8. CCL18 plays a role in promoting breast cancer, colon cancer, and squamous cell carcinoma metastasis [37], and CCR8 + Tregs are highly suppressive cells within the tumor [48]. We observed a correlation between CCL18 expression and the CTL-3 exhausted signature score, suggesting that CCL18-CCR8 axis also plays an immunosuppressive role in ccRCC bone metastases.

MSCs are critical in modulating the tumor microenvironment and MSC-derived factors affect disease progression in prostate bone metastasis [75] as well as in metastatic breast cancer [76]. MSCs segregated into two subsets, including MSC-1 that was enriched in normal samples while MSC-2 was enriched in the bone metastatic samples. The MSC-2 population was characterized by an enhanced EMT program and CAF phenotype (Fig. 4G, H). These changes imply that this subpopulation of MSCs in ccRCC bone metastases may be similar to CAFs seen in other cancers [77]. CAFs are a key component of the TME; they can modulate cancer metastasis through the remodeling of the extracellular matrix (ECM) and production of growth factors and influence angiogenesis and immune response. Indeed, we observed expression of IL6, IL8, VEGFA, and TGFB1, as well as collagen-associated genes (COL6A2, COL3A1, COL4A1, COL4A2) (Fig. 4E), which is reported in CAFs [60].

Tumor cells can exploit certain aspects of the bone ME for homing, maintenance, and growth [1, 4]. In the osteolytic bone metastases of patients with bone metastatic ccRCC, bone resorption mediated by osteoclasts is preferentially activated over bone formation [4]. The RANK-RANKL axis is a major pathway promoting osteoclast-mediated bone resorption through favoring osteoclast differentiation and maturation [78]. Here, we demonstrated that this mechanism is increased in bone metastatic ccRCC (Fig. 6B–D). Furthermore, the level of OPG, with its role in bone growth and homeostasis, was significantly decreased (Fig. 6D). OSM has been shown to independently stimulate the expression of RANKL through direct contact with MSCs via OSMR [64]. Our results support the relationship of the OSM-OSMR axis (Additional file 2: Fig. S7B) in promoting an osteolytic microenvironment as the tumor-associated MSC-2 population as well as the tumor cells themselves gain expression of OSMR, thereby favoring aberrant osteoclast formation and differentiation.

Although our analysis presents a good representation of immune and stroma cells in the ccRCC primary and bone metastatic niche, it is important to consider a few potential limitations of our study. One of the main limitations is lack of validation in separate patient samples. Although we performed functional interpretation and protein validation of certain cell types using the same patient cohort, validation in independent datasets will be necessary to further substantiate these findings. Furthermore, the analysis of survival curves using bulk RNA-seq gene expression data can be challenging due to the potential confounding factors, such as age, gender, treatment status, genetics, risk group, and technical biases within large bulk RNA-seq cohorts. However, despite these challenges, we were able to identify significant differences that we believe might have a critical clinical implication for understanding how immune and stroma cells impact ccRCC survival.

## Conclusions

Our single-cell transcriptomic analysis of ccRCC prim[ary and bone metastatic tumors revealed the dynamics of immune and stroma cell remodeling during tumor progression and metastasis. We found that the bone metastatic niche is markedly immune suppressive with increased exhausted CD8 + cytotoxic T cells, T regulatory cells, and TAMs. Within the TAMs, the TREM2 + SPP1 + subset was notably enriched in bone metastatic lesions and was associated with worse patient survival, implicating a potential role in metastatic progression. Additionally, our study captured a tumor-associated mesenchymal stromal cell population (TA-MSC), which is transcriptionally similar to CAFs, which appears to contribute to the epithelial-to-mesenchymal transition and to bone remodeling. Overall, this comprehensive analysis offers valuable insights into the biology of ccRCC bone metastases and highlights potential therapeutic avenues targeting the diverse cellular constituents of the tumor microenvironment.

### Supplementary Information

**Additional file 1:** **Table S1.** Basic information of sample cohort. **Table S2.** Data quality control metrics. **Table S3.** Patient sample groups and cell frequency. **Table S4.** Signature genes for cell annotations. **Table S5.** List of gene signature score. **Table S6.** Significant ligand and receptor pairs. **Table S7.** Cox-regression survival result. **Table S8.** Flow cytometry panels for myeloid and T cells.

**Additional file 2:** **Fig S1.** Overview of immune and stromal cell landscape in ccRCC bone metastasis. **Fig S2.** Distinct tumor-associated macrophage subpopulations in ccRCC bone metastasis. **Fig S3.** Sustained T cells dysfunction in ccRCC primary and bone metastatic tumors. **Fig S4.** Dysfunctional T cells correlate with Macro-2. **Fig S5.** Changes of stromal cell subpopulations in the ccRCC bone metastasis. **Fig S6.** Tumor cells heterogeneity within human ccRCC bone metastasis. **Fig S7.** Tumor-associated MSCs source to bone remodeling of ccRCC bone metastasis.

## Data Availability

The single-cell data set generated in this study is available from GEO (https://www.ncbi.nlm.nih.gov/geo) with accession GSE202813 [79]. The public data analyzed in this study are available on GEO with accessions GSE120446/GSE120221 [10], GSE178481 [11], and Single Cell Portal (SCP1288) [41]. The TCGA clear cell renal cell carcinoma (KIRC) [80] cohort was downloaded from the cBioPortal (https://www.cbioportal.org/study/clinicalData?id=kirc_tcga). The custom code and the combined data that was used in this study can be found at https://github.com/shenglinmei/ccRCC.bone.Met [81].
