# Supplementary material for: Single-cell analysis of immune and stroma cell remodeling in clear cell renal cell carcinoma primary tumors and bone metastatic lesions
Source: Genome Med. 2024 Jan 29;16:1. doi: 10.1186/s13073-023-01272-6 (PMC10823713; doi:10.1186/s13073-023-01272-6)
Supplement: Supplementary file 2 — Additional file 2: Fig S1. Overview of immune and stromal cell landscape in ccRCC bone metastasis. Fig S2. Distinct tumor-associated macrophage subpopulations in ccRCC bone metastasis. Fig S3. Sustained T cells dysfunction in ccRCC primary and bone metastatic tumors. Fig S4. Dysfunctional T cells correlate with Macro-2. Fig S5. Changes of stromal cell subpopulations in the ccRCC bone metastasis. Fig S6. Tumor cells heterogeneity within human ccRCC bone metastasis. Fig S7. Tumor-associated MSCs source to bone remodeling of ccRCC bone metastasis. [file 13073_2023_1272_MOESM2_ESM.pdf]

Supplementary Information

Additional file 2: Supplementary Materials for: Single-cell analysis of immune and stroma cell remodeling in clear cell renal cell carcinoma primary tumors and bone metastatic lesions. This file includes Supplementary Figures: Fig. S1-S7

Fig S1. Overview of immune and stromal cell landscape in ccRCC bone metastasis.

Fig S2. Distinct tumor-associated macrophage subpopulations in ccRCC bone metastasis.

Fig S3. Sustained T cells dysfunction in ccRCC primary and bone metastatic tumors.

Fig S4. Dysfunctional T cells correlate with Macro-2.

Fig S5. Changes of stromal cell subpopulations in the ccRCC bone metastasis.

Fig S6. Tumor cells heterogeneity within human ccRCC bone metastasis.

Fig S7. Tumor associated MSCs source to bone remodeling of ccRCC bone metastasis.

Fig S1

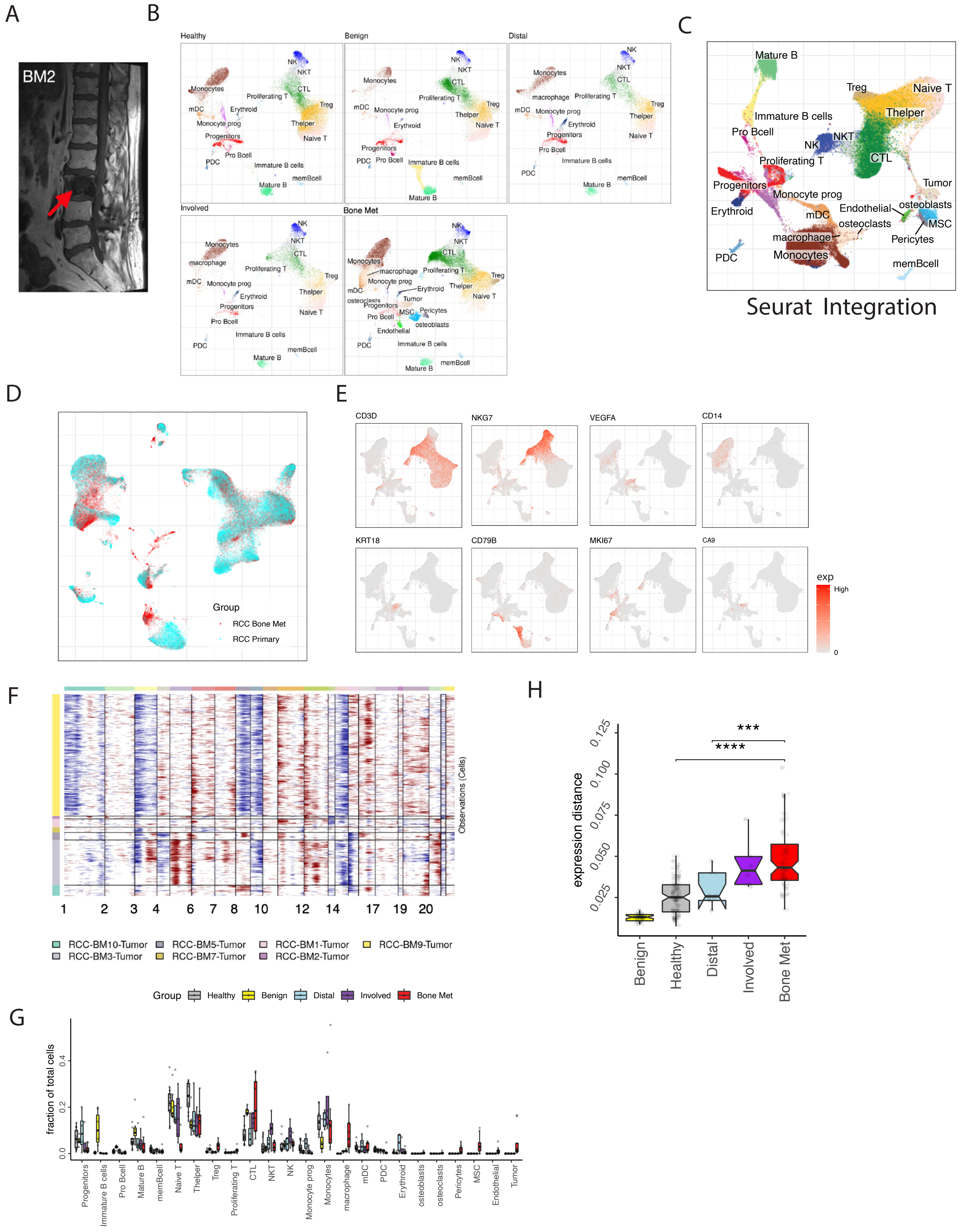

A
BM2
B
Healthy
Benign
Distal
Involved
Bone Met
C
Seurat Integration
D
Group
RCC Bone Met
RCC Primary
E
CD3D
NKG7
VEGFA
CD14
KRT18
CD79B
MKI67
CA9
exp
High
0
F
Observations (Cells)
1 2 3 4 6 7 8 10 12 14 17 19 20
RCC-BM10-Tumor
RCC-BM5-Tumor
RCC-BM1-Tumor
RCC-BM9-Tumor
RCC-BM3-Tumor
RCC-BM7-Tumor
RCC-BM2-Tumor
Group Healthy Benign Distal Involved Bone Met
G
fraction of total cells
Progenitors
Immature B cells
Pro Bcell
Mature B
memBcell
Naive T
Thelper
Treg
Proliferating T
CTL
NKT
NK
Monocyte prog
Monocytes
macrophage
mDC
PDC
Erythroid
osteoblasts
osteoclasts
Pericytes
MSC
Endothelial
Tumor
H
expression distance
***
****
Benign
Healthy
Distal
Involved
Bone Met

Fig S1. Overview of immune and stromal cell landscape in ccRCC bone metastasis.

A. Similar to Figure 1B, the sagittal T1 MRI imaging of the thoracic spine is shown for patient BM2.

B. Joint UMAP embedding showing cells from different sample fractions.

C. Seurat integration of scRNA-seq samples of all bone marrow samples (*Healthy*, *Benign*, *Involved*, *Distal* and *Bone Met*), visualized using a common UMAP embedding color coded by the cell type.

D. Integrative analysis of scRNA-seq samples visualized using a common UMAP embedding for cell sample fractions (ccRCC primary and ccRCC Bone Met tumors).

E. UMAP visualization showing expression of selected marker genes.

F. InferCNV analysis showing pronounced patients specific CNV pattern taking proximal tube cells as control (see method).

G. Comparison of relative cell abundance of major cell clusters between *Bone Met* (n=9) and different control fractions (*Healthy* n=12, *Benign* n=7, *Involved* n=4, *Distal* n=4). Statistics are accessed with two-sided Wilcoxon rank sum test (*p<0.05, ***p<0.001, Additional file 1: Table S3). For box plots, center line represents the median and box limits represent upper and lower quartiles, and whiskers depicts 1.5 × the interquartile range (IQR).

H. Boxplot showing inter-individual gene expression distances (based on Pearson correlation) within Healthy, Benign, Distal, Involved and Tumor fractions. Significance was assessed using two-sided Wilcoxon rank sum test and BH multiple testing correction (***p<0.001, **** p<0.0001).

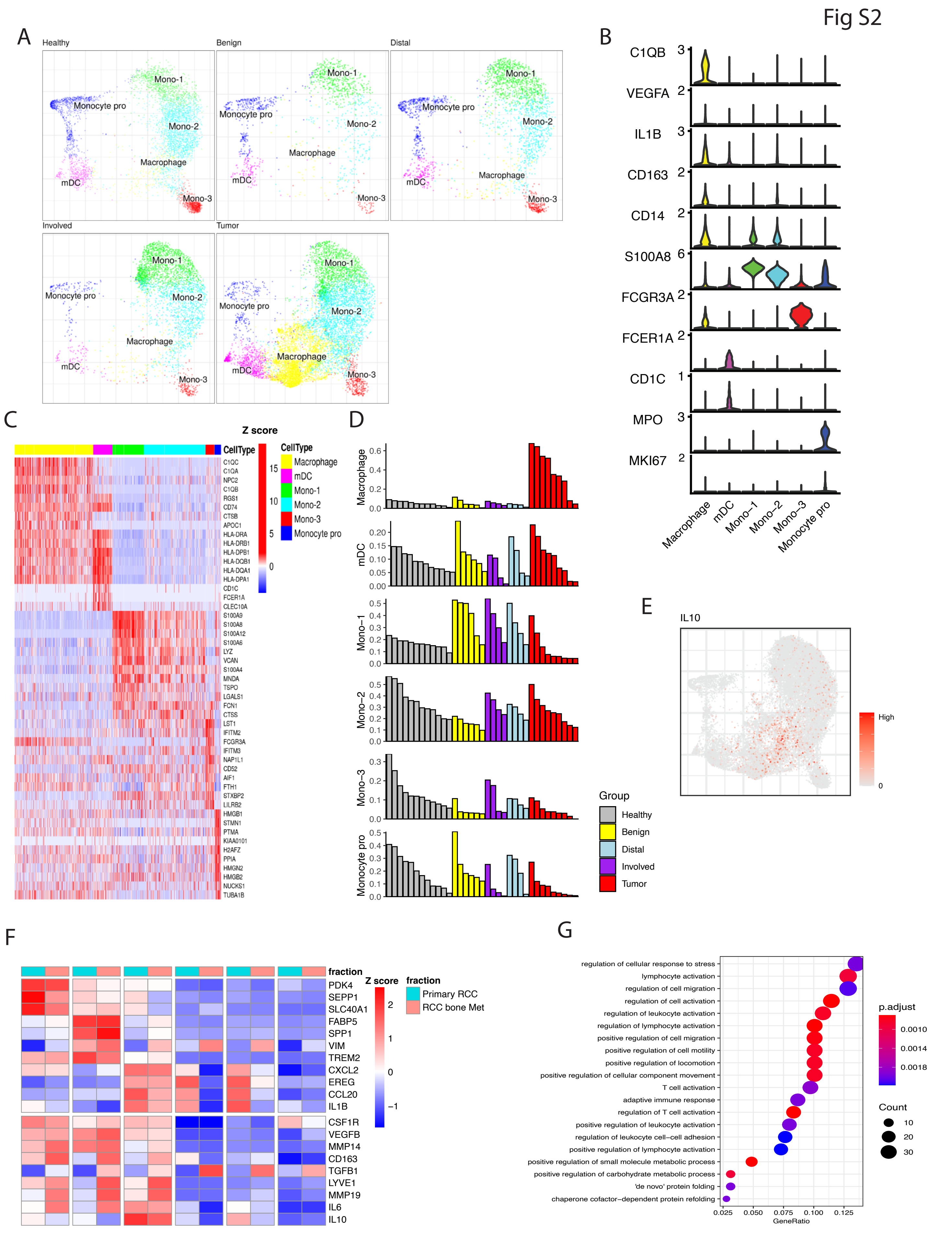

Fig S2
A
Healthy
Benign
Distal
Involved
Tumor
Mono-1
Mono-2
Mono-3
Monocyte pro
Macrophage
mDC
B
C1QB
VEGFA
IL1B
CD163
CD14
S100A8
FCGR3A
FCER1A
CD1C
MPO
MKI67
Macrophage
mDC
Mono−1
Mono−2
Mono−3
Monocyte pro
C
Z score
CellType
Macrophage
mDC
Mono-1
Mono-2
Mono-3
Monocyte pro
D
Macrophage
mDC
Mono−1
Mono−2
Mono−3
Monocyte pro
Group
Healthy
Benign
Distal
Involved
Tumor
E
IL10
High
0
F
fraction
Z score
fraction
Primary RCC
RCC bone Met
PDK4
SEPP1
SLC40A1
FABP5
SPP1
VIM
TREM2
CXCL2
EREG
CCL20
IL1B
CSF1R
VEGFB
MMP14
CD163
TGFB1
LYVE1
MMP19
IL6
IL10
G
regulation of cellular response to stress
lymphocyte activation
regulation of cell migration
regulation of cell activation
regulation of leukocyte activation
regulation of lymphocyte activation
positive regulation of cell migration
positive regulation of cell motility
positive regulation of locomotion
positive regulation of cellular component movement
T cell activation
adaptive immune response
regulation of T cell activation
positive regulation of leukocyte activation
regulation of leukocyte cell−cell adhesion
positive regulation of lymphocyte activation
positive regulation of small molecule metabolic process
positive regulation of carbohydrate metabolic process
'de novo' protein folding
chaperone cofactor−dependent protein refolding
GeneRatio
p.adjust
Count

Fig S2. Distinct tumor-associated macrophage subpopulations in ccRCC bone metastasis.

A. UMAP embedding demonstrating myeloid cell subpopulations from different sample conditions.

B. Violin plot showing representative marker gene expression of myeloid subpopulations.

C. Scaled average expression of Macrophage markers and M2 signature gene expression shown as heatmap for macrophage subpopulations.

D. Barplot illustrating the relative cell proportion of myeloid subsets in each individual patient samples.

E. IL10 expression on joint UMAP embedding.

F. Heatmap shows an overview of genes (rows) differentially expressed between myeloid subpopulations.

G. Enriched GO terms (y-axis) of top 300 upregulated genes in Macro-2 comparing *ccRCC Bone Met* versus *ccRCC Primary* tumors. The x axis shows the fraction of expressed genes from a GO term that were part of the program. Dot size indicates the number of such genes, and the color shows adjusted p-value (BH adjustment).

Fig S3

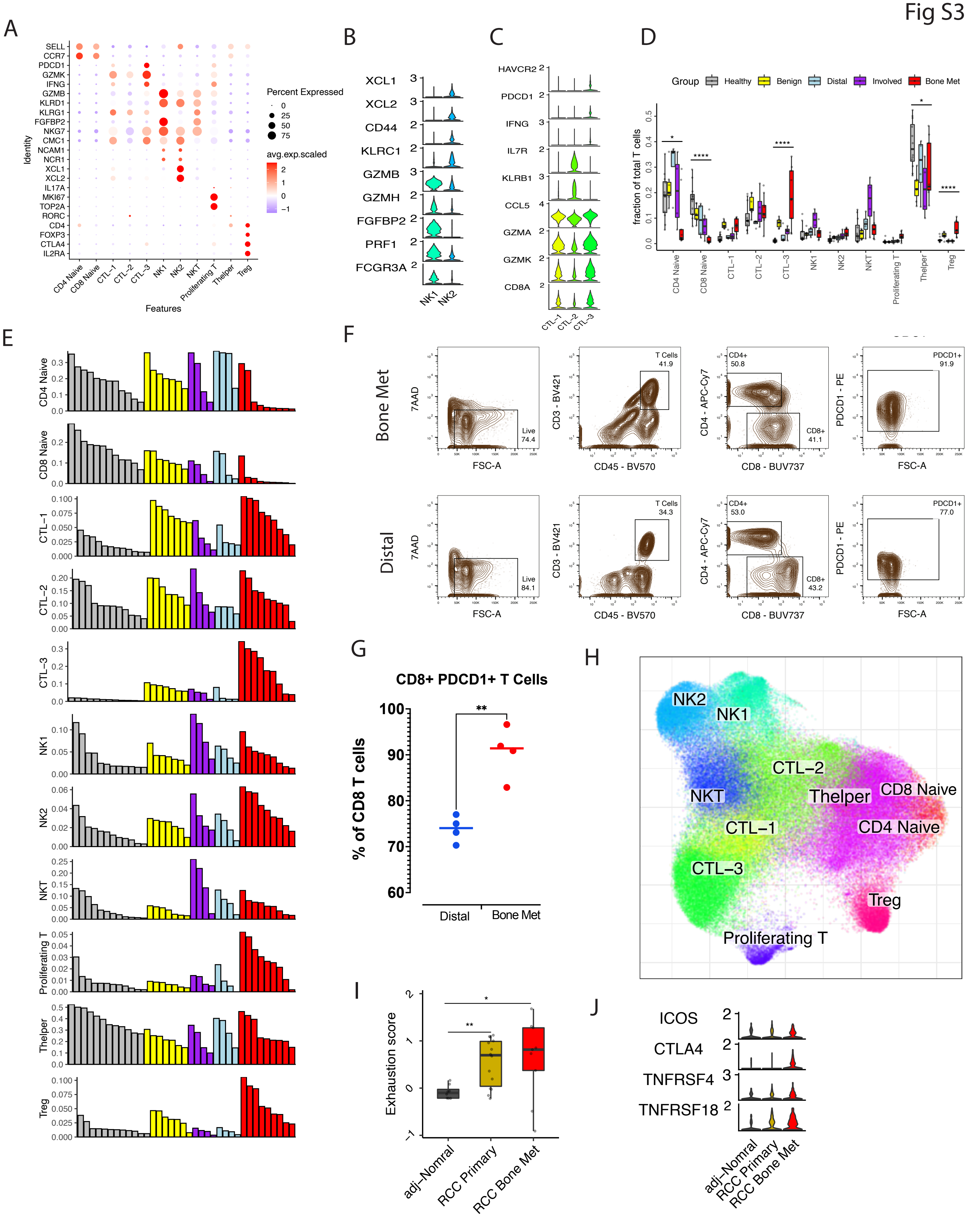
A
B
C
D
E
F
G
H
I
J
Percent Expressed
avg.exp.scaled
Features
Identity
Group
Healthy
Benign
Distal
Involved
Bone Met
fraction of total T cells
Bone Met
Distal
CD8+ PDCD1+ T Cells
% of CD8 T cells
Exhaustion score
adj-Nomral
RCC Primary
RCC Bone Met
ICOS
CTLA4
TNFRSF4
TNFRSF18
NK2
NK1
CTL–2
NKT
Thelper
CD8 Naive
CD4 Naive
CTL–1
CTL–3
Treg
Proliferating T

Fig S3. Sustained T cells dysfunction in ccRCC primary and bone metastatic tumors.

A. Dot plots showing representative marker gene expression across different T cell subsets. The color represents scaled average expression of marker genes in each cell type, and the size indicates the proportion of cells expressing marker genes.

B. Violin plot showing selected gene expression in NK1 and NK2.

C. Violin plot showing selected gene expression for three CTLs subpopulations.

D. Boxplots showing the proportions of T cell subsets divided by the total T cells across *Bone Met* (n=9) and different control fractions (*Healthy* n=12, *Benign* n=7, *Involved* n=4, *Distal* n=4). Statistics are accessed with two-sided Wilcoxon rank sum test and BH multiple testing correction. (*$p<0.05$, ***$p<0.001$, Additional file 1: Table S3).

E. Barplot illustrating the relative cell proportion of T cell subsets in each individual patient samples.

F. Gating strategy applied for flow cytometry of PDCD1+CD8+ T cells for *Bone Met* (top) and *Distal* (bottom) BM tissue.

G. Boxplot showing the percent of PDCD1+CD8+ T cells in *Distal* (n=4) and *Bone Met* (n=4) tissue by flow cytometry. Statistical significance determined using two-sided t-test (*$p<0.05$).

H. UMAP joint embedding of T and NK cell populations from ccRCC Bone Met and ccRCC primary tumors.

I. Boxplot showing exhaustion score in CTL-3 across *RCC Bone Met* (n=9) with *RCC Primary* (n=14) and *adjacent normal* tissue (n=9). Significance was assessed using two-sided Wilcoxon rank sum test (*$p<0.05$, **$p<0.01$).

I. Immune suppressive gene expression in Tregs shown as violin plot.

For box plots, center line represents the median and box limits represent upper and lower quartiles, and whiskers depicts 1.5 × the interquartile range (IQR).

Fig S4

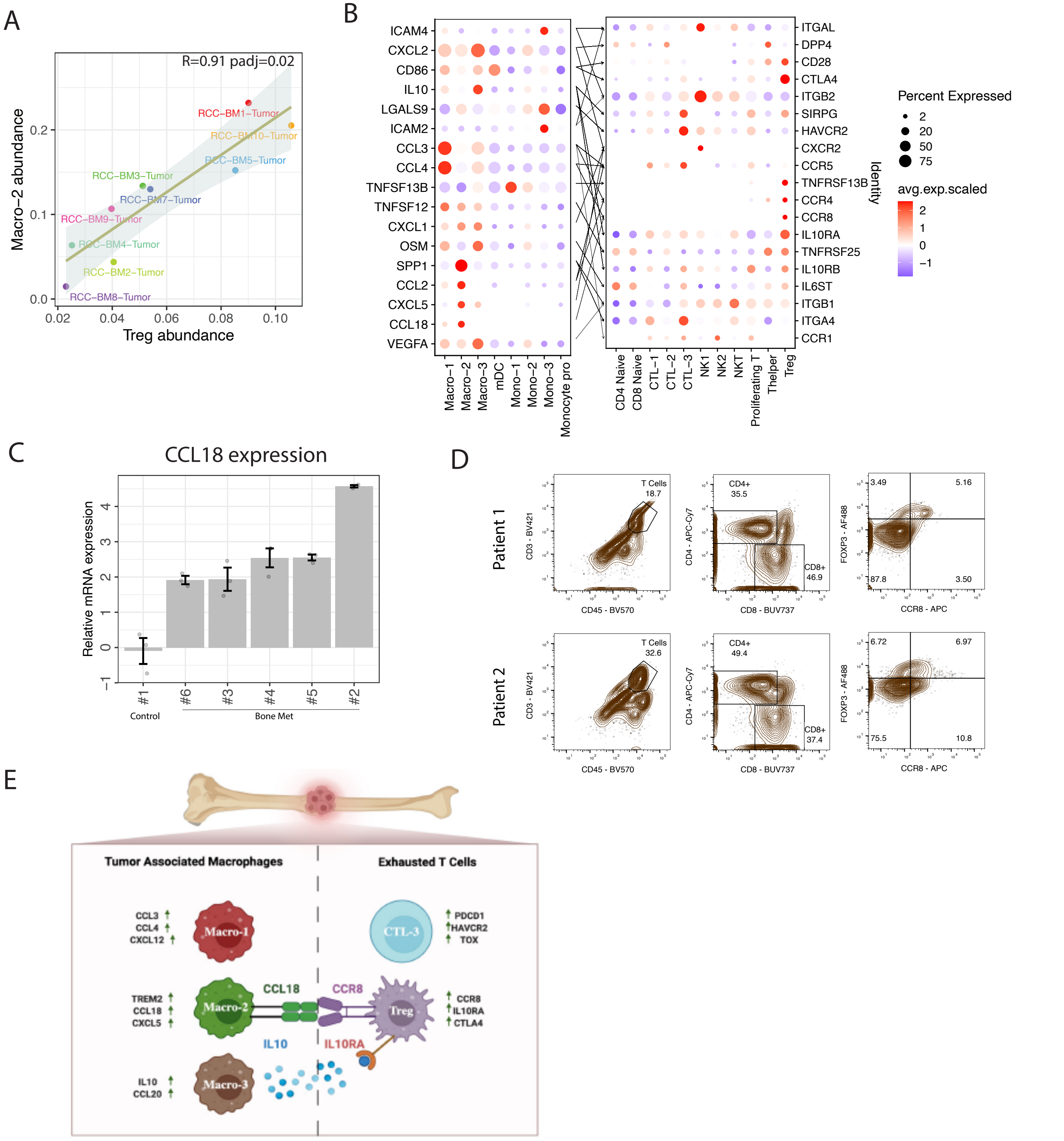

A
R=0.91 padj=0.02
RCC−BM1−Tumor
RCC−BM10−Tumor
RCC−BM5−Tumor
RCC−BM3−Tumor
RCC−BM7−Tumor
RCC−BM9−Tumor
RCC−BM4−Tumor
RCC−BM2−Tumor
RCC−BM8−Tumor
Macro−2 abundance
Treg abundance
B
ICAM4
CXCL2
CD86
IL10
LGALS9
ICAM2
CCL3
CCL4
TNFSF13B
TNFSF12
CXCL1
OSM
SPP1
CCL2
CXCL5
CCL18
VEGFA
Macro−1
Macro−2
Macro−3
mDC
Mono−1
Mono−2
Mono−3
Monocyte pro
ITGAL
DPP4
CD28
CTLA4
ITGB2
SIRPG
HAVCR2
CXCR2
CCR5
TNFRSF13B
CCR4
CCR8
IL10RA
TNFRSF25
IL10RB
IL6ST
ITGB1
ITGA4
CCR1
CD4 Naive
CD8 Naive
CTL−1
CTL−2
CTL−3
NK1
NK2
NKT
Proliferating T
Thelper
Treg
Identity
Percent Expressed
2
20
50
75
avg.exp.scaled
2
1
0
−1
C
CCL18 expression
Relative mRNA expression
#1
#6
#3
#4
#5
#2
Control
Bone Met
D
Patient 1
Patient 2
T Cells
18.7
32.6
CD4+
35.5
49.4
CD8+
46.9
37.4
3.49
5.16
87.8
3.50
6.72
6.97
75.5
10.8
CD3 - BV421
CD45 - BV570
CD4 - APC-Cy7
CD8 - BUV737
FOXP3 - AF488
CCR8 - APC
E
Tumor Associated Macrophages
Exhausted T Cells
CCL3
CCL4
CXCL12
Macro-1
CTL-3
PDCD1
HAVCR2
TOX
TREM2
CCL18
CXCL5
Macro-2
CCL18
CCR8
Treg
CCR8
IL10RA
CTLA4
IL10
IL10RA
IL10
CCL20
Macro-3

Fig S4. Dysfunctional T cells correlate with Macro-2.

A. Scatter plot showing the correlation between Macro-2 abundance and Treg abundance. Each dot represents a sample. Pearson linear correlation estimate, and p-values are shown. The error band indicates 95% confidence interval.

B. Bubble heatmap showing expression of ligand (left: myeloid cell) and receptor (right: T cell subsets) pairs. Dot size indicates expression ratio, colored represents average gene expression.

C. Barplot showing relative mRNA expression (log fold change) of CCL18 in TREM2+ SPP1+ macrophages by RT-qPCR. Data are expressed using the $2^{-\Delta\Delta Ct}$ method. Gene expression levels were normalized to the Benign control.

D. Gating strategy for enrichment for CCR8+ Tregs in Bone Met patients. Labels above the flow plots refer to the parent population in the percentages are of the parent gate.

E. Schematic illustration of immunosuppressive microenvironment featuring increased tumor-associated macrophages (TAM) with distinct transcriptional states, exhausted CD8+ T cells, and Tregs.

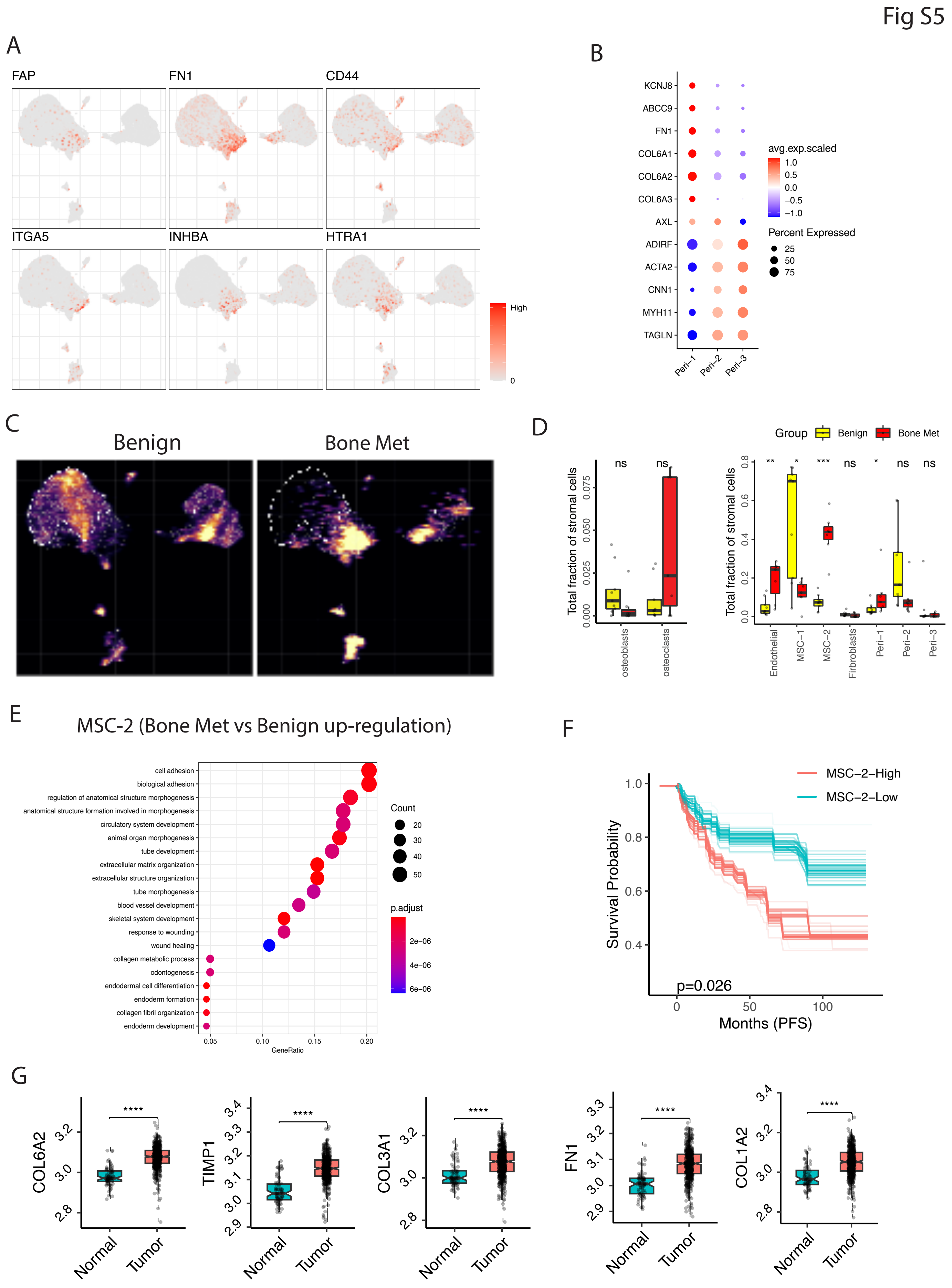
Fig S5
A
FAP
FN1
CD44
ITGA5
INHBA
HTRA1
High
0
B
KCNJ8
ABCC9
FN1
COL6A1
COL6A2
COL6A3
AXL
ADIRF
ACTA2
CNN1
MYH11
TAGLN
Peri−1
Peri−2
Peri−3
avg.exp.scaled
Percent Expressed
C
Benign
Bone Met
D
Group
Benign
Bone Met
Total fraction of stromal cells
osteoblasts
osteoclasts
Endothelial
MSC−1
MSC−2
Firbroblasts
Peri−1
Peri−2
Peri−3
E
MSC-2 (Bone Met vs Benign up-regulation)
cell adhesion
biological adhesion
regulation of anatomical structure morphogenesis
anatomical structure formation involved in morphogenesis
circulatory system development
animal organ morphogenesis
tube development
extracellular matrix organization
extracellular structure organization
tube morphogenesis
blood vessel development
skeletal system development
response to wounding
wound healing
collagen metabolic process
odontogenesis
endodermal cell differentiation
endoderm formation
collagen fibril organization
endoderm development
Count
p.adjust
GeneRatio
F
MSC−2−High
MSC−2−Low
Survival Probability
p=0.026
Months (PFS)
G
COL6A2
TIMP1
COL3A1
FN1
COL1A2
Normal
Tumor

Fig S5. Changes of stromal cell subpopulations in the ccRCC bone metastasis.

A. Selected stroma marker gene expression on joint UMAP embedding.

B. Dot plots showing marker gene expression across different pericytes subpopulations.

C. Shifts in the stroma cell populations visualized as cell density on the joint UMAP embedding. Average density of *Benign* and *Bone Met* samples are shown, Brighter colors correspond to denser regions.

D. Boxplot representing the proportion of stroma cell subsets in *Bone Met* (n=9) and *Benign* (n=9) fraction. Significance was assessed using two-sided Wilcoxon rank sum test (*p<0.05, **p<0.01).

E. Barplot illustrating the relative cell proportion of MSC-1 and MSC-2 in each individual patient samples.

F. Enriched GO terms (y-axis) of top 300 upregulated genes in MSC-1 and MSC-2 comparing *Bone Met* versus *Benign* condition. The x axis shows the fraction of expressed genes from a GO term that were part of the program. Dot size indicates the number of such genes, and the color shows adjusted p-value (BH adjustment).

G. Boxplot representing Macro-2 signature gene expression in TCGA KIRC tumor (n=533) and adjacent normal (n=73) bulk RNA samples. Statistics are accessed with two-sided Wilcoxon rank sum test.

H. Similar with Fig. 4J, Kaplan–Meier curves showing ccRCC samples with higher MSC-2 signature gene expression have worse progression-free survival (n=435) in TCGA KIRC data. P value was evaluated using Log-rank test. Bootstrap resampling was performed on signature genes and p-value was calculated using the 95% reproducibility power p-value (see Methods).

For box plots, center line represents the median and box limits represent upper and lower quartiles, and whiskers depicts 1.5 × the interquartile range (IQR).

Fig S6

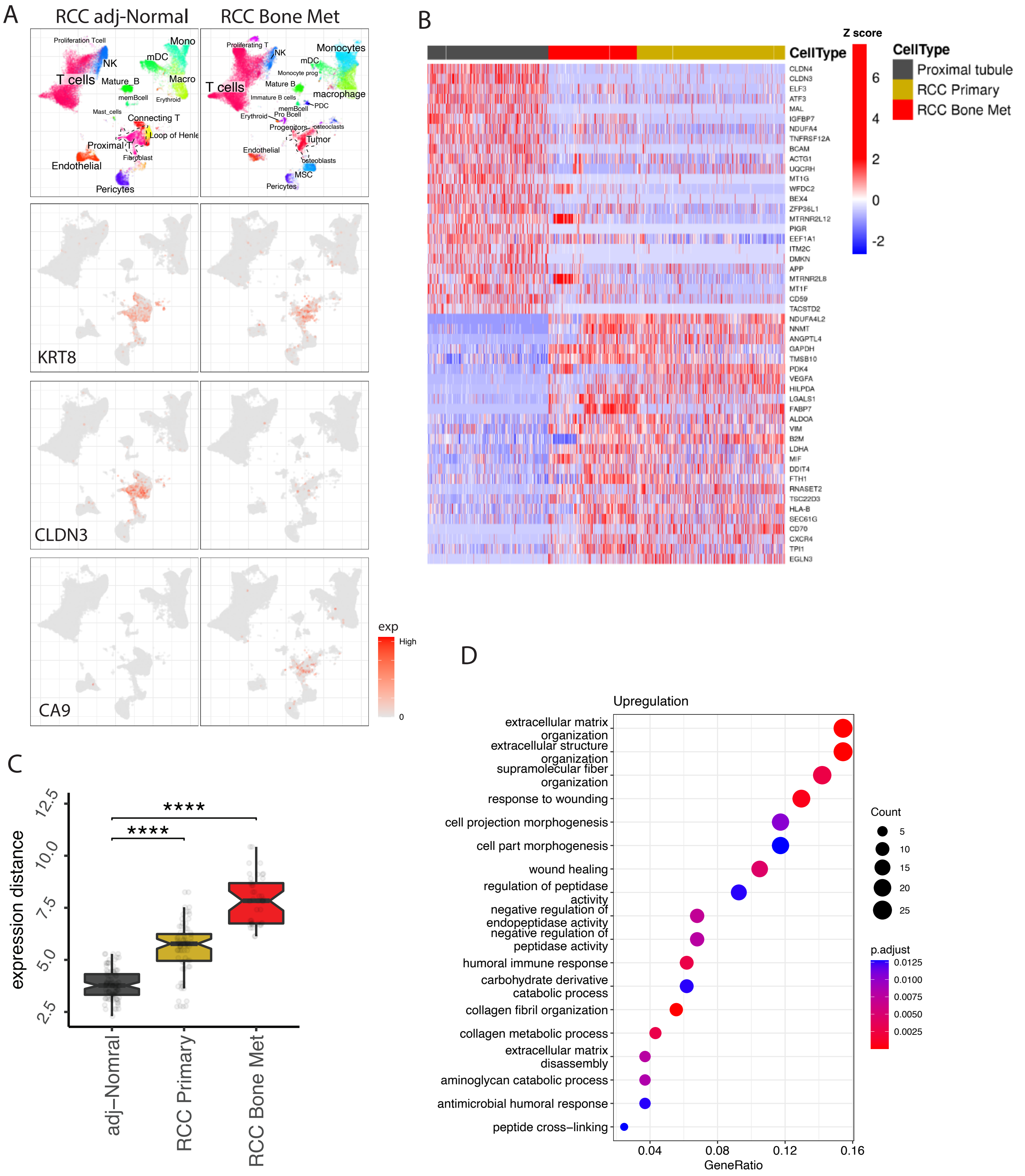

A
RCC adj-Normal
RCC Bone Met
Proliferation Tcell
Mono
NK
mDC
T cells
Mature_B
Macro
memBcell
Erythroid
Mast_cells
Connecting T
Loop of Henle
Proximal T
Fibroblast
Endothelial
Pericytes
Proliferating T
Monocytes
Monocyte prog
Mature B
macrophage
Immature B cells
PDC
Pro Bcell
Progenitors
osteoclasts
Tumor
osteoblasts
MSC
KRT8
CLDN3
CA9
exp
High
0
B
Z score
CellType
Proximal tubule
RCC Primary
RCC Bone Met
CLDN4
CLDN3
ELF3
ATF3
MAL
IGFBP7
NDUFA4
TNFRSF12A
BCAM
ACTG1
UQCRH
MT1G
WFDC2
BEX4
ZFP36L1
MTRNR2L12
PIGR
EEF1A1
ITM2C
DMKN
APP
MTRNR2L8
MT1F
CD59
TACSTD2
NDUFA4L2
NNMT
ANGPTL4
GAPDH
TMSB10
PDK4
VEGFA
HILPDA
LGALS1
FABP7
ALDOA
VIM
B2M
LDHA
MIF
DDIT4
FTH1
RNASET2
TSC22D3
HLA-B
SEC61G
CD70
CXCR4
TPI1
EGLN3
6
4
2
0
-2
C
expression distance
****
****
12.5
10.0
7.5
5.0
2.5
adj–Nomral
RCC Primary
RCC Bone Met
D
Upregulation
extracellular matrix organization
extracellular structure organization
supramolecular fiber organization
response to wounding
cell projection morphogenesis
cell part morphogenesis
wound healing
regulation of peptidase activity
negative regulation of endopeptidase activity
negative regulation of peptidase activity
humoral immune response
carbohydrate derivative catabolic process
collagen fibril organization
collagen metabolic process
extracellular matrix disassembly
aminoglycan catabolic process
antimicrobial humoral response
peptide cross–linking
0.04
0.08
0.12
0.16
GeneRatio
Count
5
10
15
20
25
p.adjust
0.0125
0.0100
0.0075
0.0050
0.0025

Fig S6. Tumor cells heterogeneity within human ccRCC bone metastasis

A. Integrative analysis of scRNA-seq samples from *adjacent normal kidney* tissue and *Bone Met* tumors, visualized using a common UMAP embedding (top). UMAP visualization of representative marker gene expression in *adjacent normal kidney* tissue and *Bone Met* tumors (bottom).

B. Heatmap showing differential expressed genes comparing proximal tubule of the normal adjacent kidney tissue, primary ccRCC tumor and the ccRCC bone metastatic tumor.

C. Expression distance comparing proximal tubule of the *normal adjacent kidney* tissue, *ccRCC primary* tumor and the *ccRCC Bone Met*. Significance was assessed using two-sided Wilcoxon rank sum test (****p<0.0001).

D. Enriched GO terms (y-axis) of top 300 upregulated genes in *ccRCC Bone Met* tumor cells. The x axis shows the fraction of expressed genes from a GO term that were part of the program. Dot size indicates the number of such genes, and the color shows adjusted p-value (BH adjustment).

Fig S7

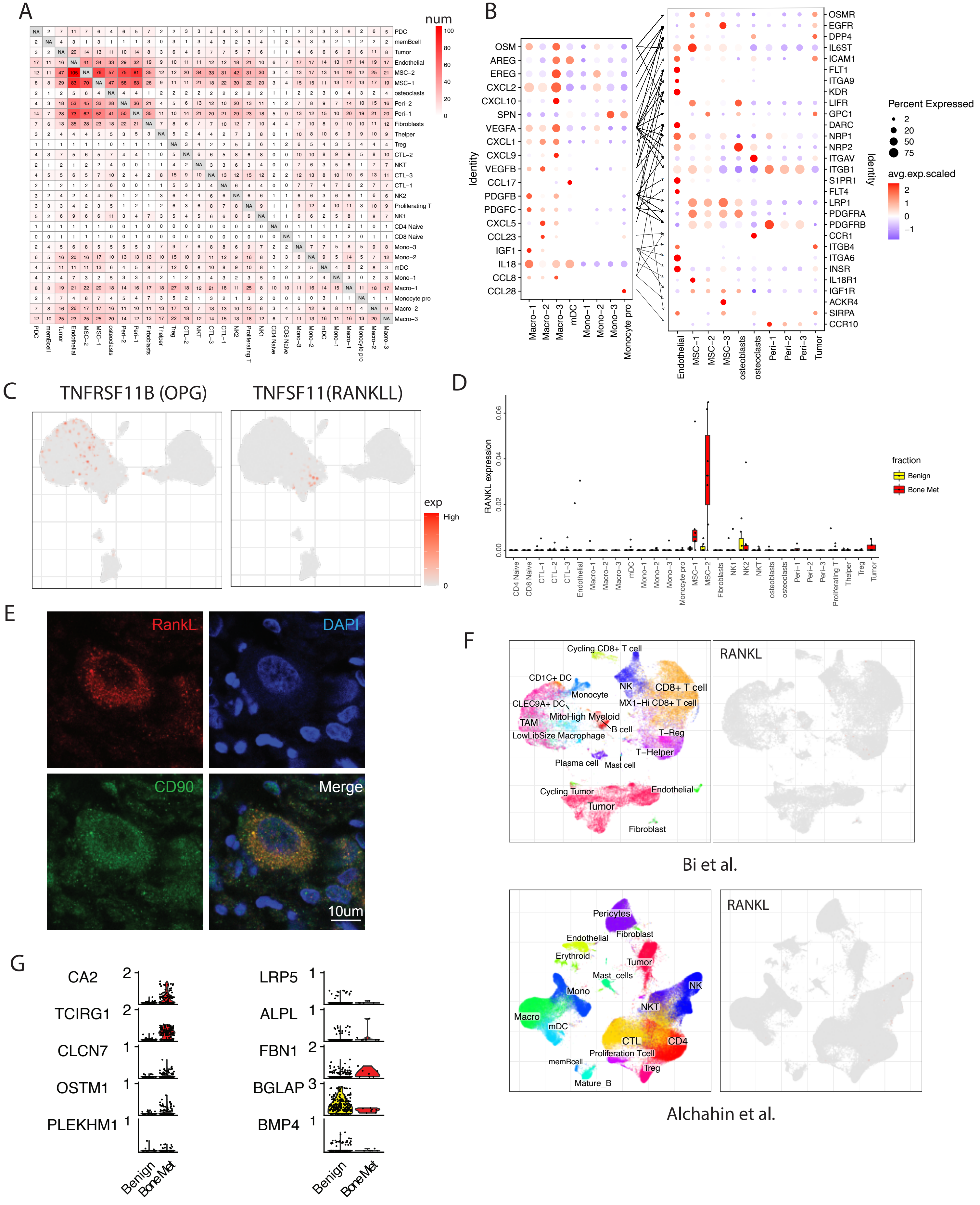
A
num
B
Percent Expressed
avg.exp.scaled
Identity
C
TNFRSF11B (OPG)
TNFSF11(RANKLL)
exp
High
D
RANKL expression
fraction
Benign
Bone Met
E
RankL
DAPI
CD90
Merge
10um
F
RANKL
Bi et al.
Alchahin et al.
G
CA2
TCIRG1
CLCN7
OSTM1
PLEKHM1
LRP5
ALPL
FBN1
BGLAP
BMP4
Benign
BoneMet

Fig S7. Tumor associated MSCs source to bone remodeling of ccRCC bone metastasis

A. Table displaying the number of interactions between the different subpopulations present in human ccRCC bone metastasis tumors (Additional file 1: Table S6).

B. Bubble heatmap showing expression of ligand (left: myeloid cell subsets) and receptor (right: stromal subsets) pairs in different myeloid and stromal subsets. Dot size indicates expression ratio, colored represents average gene expression.

C. UMAP visualization of OPG and RANKL expression on stroma cells.

D. Boxplot representing RANKL expression in human ccRCC bone metastasis TME. Average gene expression was used, each dot represents a sample. Significance was assessed using a two-sided Wilcoxon rank sum test.

E. Immunostaining in tissue from bone metastatic ccRCC stained for RANKL, MSC-2 specific marker CD90 and DAPI.

F. UMAP embedding showing the major cell types within primary ccRCC patients (left) and RANK expression (right) from two independent scRNA-seq datasets.

G. Violin plot showing expression of differential expressed genes between Tumor and Benign condition in osteoclast (left) and osteoblast (right).
